# Supplementary material for: Diverse Sensory Repertoire of Paralogous Chemoreceptors Tlp2, Tlp3, and Tlp4 in Campylobacter jejuni
Source: Microbiol Spectr. 2022 Nov 14;10(6):e03646-22. doi: 10.1128/spectrum.03646-22 (PMC9769880; doi:10.1128/spectrum.03646-22)
Supplement: Supplemental file 1 — Fig. S1 to S9, legends for Videos S1 and S2, and Tables S1 to S8. Download spectrum.03646-22-s0001.pdf, PDF file, 2.7 MB [file spectrum.03646-22-s0001.pdf]

Diverse sensory repertoire of paralogous chemoreceptors Tlp2, Tlp3 and Tlp4 in *Campylobacter jejuni*

Taha[1ς], Bassam A. Elgamoudi[1ς], Ekaterina P. Andrianova[2], Thomas Haselhorst[1], Christopher J. Day[1], Lauren Hartley-Tassell[1], Rebecca M. King[1], Tahria Najnin[1], Igor B. Zhulin[2] & Victoria Korolik[1,3*]

[1]Institute for Glycomics, Griffith University, Gold Coast campus, QLD 4222, Australia.

[2]Department of Microbiology and Translational Data Analytics Institute, The Ohio State University, OH 43210, USA

[3]School of Pharmacy and Medical Science, Griffith University, Gold Coast campus, QLD 4222, Australia.

Griffith University, Gold Coast campus, QLD 4222, Australia.

[ς]These authors have contributed equally to this work.

*Corresponding author. Email: v.korolik@griffith.edu.au

SUPPORTING INFORMATION

Figure S1. Protein sequence alignment showing 30% identity and signalling domain completely identical across Tlp2, Tlp3 and Tlp4.

```
Tlp3 YP_002344933.1   MLKITKIKRKIMNSIKIKLSLIANLIAIFALIVLGIVSFYFTKTSLYESTLKNQTDLLKV
Tlp2 YP_002343604.1   M-----------KSVKLKVSLIANLIAVVCLIILGVVTFIFVKQAIFHEVVNAEINYVKT
Tlp4 YP_002343704.1   MQSINSGKS---VGISAKLTLWVGILVVLILAITSAISYFDSRNNTYELLKDTQLKTMQD
                      *            .:. *::* ..::.:. * : . :::   :   :.   . : . ::

Tlp3 YP_002344933.1   TQSTVEDFRSTNQSFTRALEKDIANLPYQSLITEENIINNVGPILKYYRHSINALNVYLG
Tlp2 YP_002343604.1   AKNSIESFKARNSLALESLAKSILKHPIEQLDSQDALMHYVGKDLKNFRDAGRFLAVYIA
Tlp4 YP_002343704.1   VDAFFKSYAMSKRNGIQILANELTNRPDMS---DEELINLI-KVIKKVND---YDLVYVG
                      ..  .:.:   :    . * :.: : *  .   :: ::: :   :*  ..      **:.

Tlp3 YP_002344933.1   LNNGKVLLSQKSNDAKMPEL-----RDDLDIKTKDWYQEALKTNDIFVTPAYLDTVLKQY
Tlp2 YP_002343604.1   QPNGELVVSDPDSDAKNLDFGTYGKADNYDARTREYYIEAVKTNKLYITPSYIDVTTNLP
Tlp4 YP_002343704.1   FDNTG---KNYQSDDQILDL-----SKGYDTKNRPWYKAAKEAKKLIVTEPYKSAASGEV
                        *     .: ..* :  ::      .. * :.: :*  * :::.: :* .* ...

Tlp3 YP_002344933.1   VITYSKAIY-KDGKIIGVLGVDIPSEDLQNLVAKTPGN----TFLFDQKNKIFAATNKEL
Tlp2 YP_002343604.1   CFTYSIPLY-KDGKFIGVLAVDILAADLQAEFENLPGR----TFVFDEENKVFVSTDKAL
Tlp4 YP_002343704.1   GLTYAAPFYDRNGNFRGVVGGDYDLANFSTNVLTVGKSDNTFTEVLDSEGTILFNDEVAK
                       :**: .:* ::*:: **:. *    ::.  . .        * ::*.:..::   :

Tlp3 YP_002344933.1   LNPSIDHSP-VLNAYKLNGDN------NFFSYKL--NNEERLGACTKVF--AYTACITES
Tlp2 YP_002343604.1   LQKGYDISA-IANLAKTKEDL------EPFEYTRPKDGNERFAVCTKVS-GIYTACVGEP
Tlp4 YP_002343704.1   ILTKTELSINIANAIKANPALIDPRNQDTLFTAKDHQGVDYAIMCNSAFNPLFRICTITE
                      :    : *  : *  * :         : :      :. :    *...    :  *

Tlp3 YP_002344933.1   ADIINKPIYKAAFIQAIVVIIVVVFSVILLYFIVSKYLSPLAAIQTGLTSFFDFINYKTK
Tlp2 YP_002343604.1   IEQIEAPVYKIAFIQTAIVIFTSIISVILLYFIVSKYLSPLAAIQTGLTSFFDFINYKTK
Tlp4 YP_002343704.1   NKVYTEAVNSILMKQVIVGIIAIIIALILIRFLISRSLSPLAAIQTGLTSFFDFINYKTK
                       .    .: .  : *. : *:. ::::**: *::*: ***********************

Tlp3 YP_002344933.1   NVSTIEVKSNDEFGQISNAINENILATKRGLEQDNQAVKESVQTVSVVEGGNLTARITAN
Tlp2 YP_002343604.1   NVSTIEVKSNDEFGQISNAINENILATKRGLEQDNQAVKESVQTVSVVEGGNLTARITAN
Tlp4 YP_002343704.1   NVSTIEVKSNDEFGQISNAINENILATKRGLEQDNQAVKESVQTVSVVEGGNLTARITAN
                      ************************************************************

Tlp3 YP_002344933.1   PRNPQLIELKNVLNKLLDVLQARVGSDMNAIHKIFEEYKSLDFRNKLENASGSVELTTNA
Tlp2 YP_002343604.1   PRNPQLIELKNVLNKLLDVLQARVGSDMNAIHKIFEEYKSLDFRNKLENASGSVELTTNA
Tlp4 YP_002343704.1   PRNPQLIELKNVLNKLLDVLQARVGSDMNAIHKIFEEYKSLDFRNKLENASGSVELTTNA
                      ************************************************************

Tlp3 YP_002344933.1   LGDEIVKMLKQSSDFANALANESGKLQTAVQSLTTSSNSQAQSLEETAAALEEITSSMQN
Tlp2 YP_002343604.1   LGDEIVKMLKQSSDFANALANESGKLQTAVQSLTTSSNSQAQSLEETAAALEEITSSMQN
Tlp4 YP_002343704.1   LGDEIVKMLKQSSDFANALANESGKLQTAVQSLTTSSNSQAQSLEETAAALEEITSSMQN
                      ************************************************************

Tlp3 YP_002344933.1   VSVKTSDVITQSEEIKNVTGIIGDIADQINLLALNAAIEAARAGEHGRGFAVVADEVRKL
Tlp2 YP_002343604.1   VSVKTSDVITQSEEIKNVTGIIGDIADQINLLALNAAIEAARAGEHGRGFAVVADEVRKL
Tlp4 YP_002343704.1   VSVKTSDVITQSEEIKNVTGIIGDIADQINLLALNAAIEAARAGEHGRGFAVVADEVRKL
                      ************************************************************

Tlp3 YP_002344933.1   AERTQKSLSEIEANTNLLVQSINDMAESIKEQTAGITQINDSVAQIDQTTKDNVEIANES
Tlp2 YP_002343604.1   AERTQKSLSEIEANTNLLVQSINDMAESIKEQTAGITQINDSVAQIDQTTKDNVEIANES
Tlp4 YP_002343704.1   AERTQKSLSEIEANTNLLVQSINDMAESIKEQTAGITQINDSVAQIDQTTKDNVEIANES
                      ************************************************************

Tlp3 YP_002344933.1   AIISSTVSDIANNILEDVKKKRF
Tlp2 YP_002343604.1   AIISSTVSDIANNILEDVKKKRF
Tlp4 YP_002343704.1   AIISSTVSDIANNILEDVKKKRF
                      ***********************
```

Figure S2. Phylogenetic tree containing dCache_1 containing chemoreceptors from representative genomes of *Campylobacterota* phylum. Chemoreceptors from *C. jejuni* 11168-O used throughout this study are shown in bold. This figure is also available as a scalable pdf.

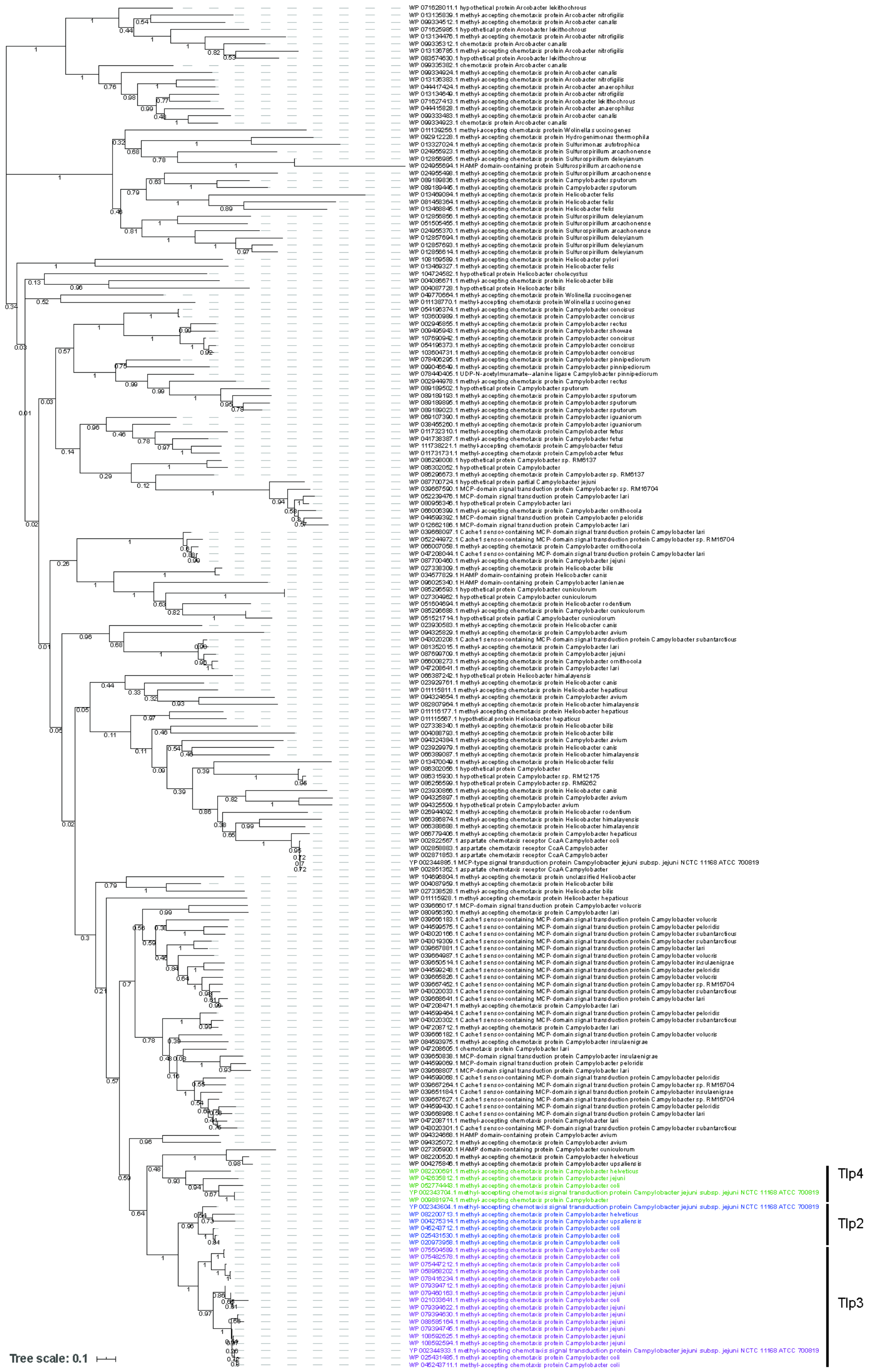

Figure S3. Relationship between dCache_1 domains from *Campylobacterota* chemoreceptors. *C. jejuni* 11168-O chemoreceptors are shown in bold. Clades containing Tlp2, Tlp3 and Tlp4 colored in blue, purple and green, respectively. Tlp1-like clade is shown in orange. Conserved amino acid recognition motif is highlighted in grey. Amino acid numeration is based on the Tlp3 protein sequence. Bootstrap support values greater than 50% are shown. This figure is also available as a scalable pdf.

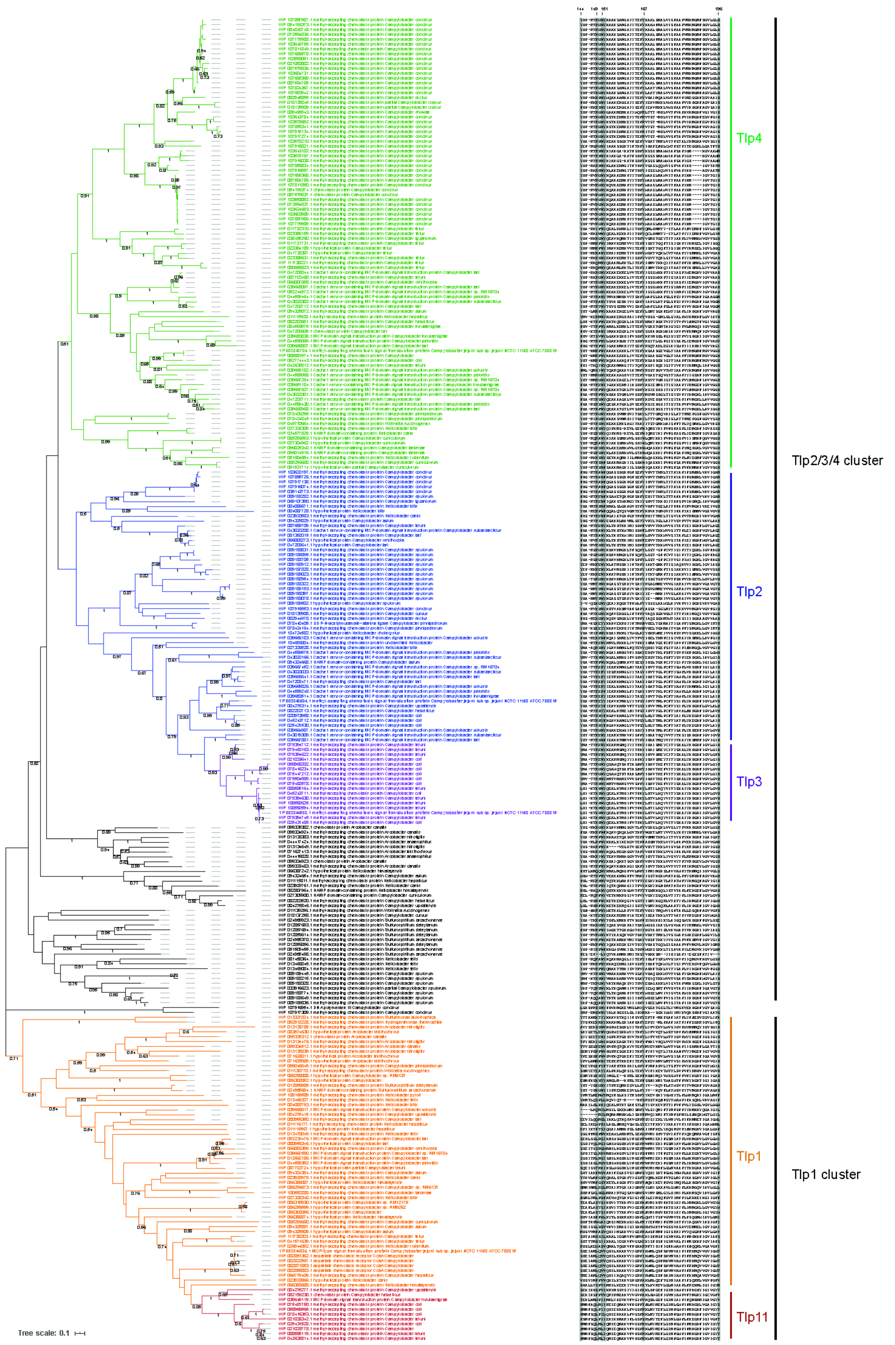

Figure S4. Phylogenetic tree of MCP signal domains from dCache_1 containing chemoreceptors. *C. jejuni* 11168-O chemoreceptors are shown in bold. Sequences are coloured according to clustering of the respective sensing domain on dCache_1 domain phylogenetic tree (Figure S3). Bootstrap percentage values greater than 40 are shown. This figure is also available as a scalable pdf.

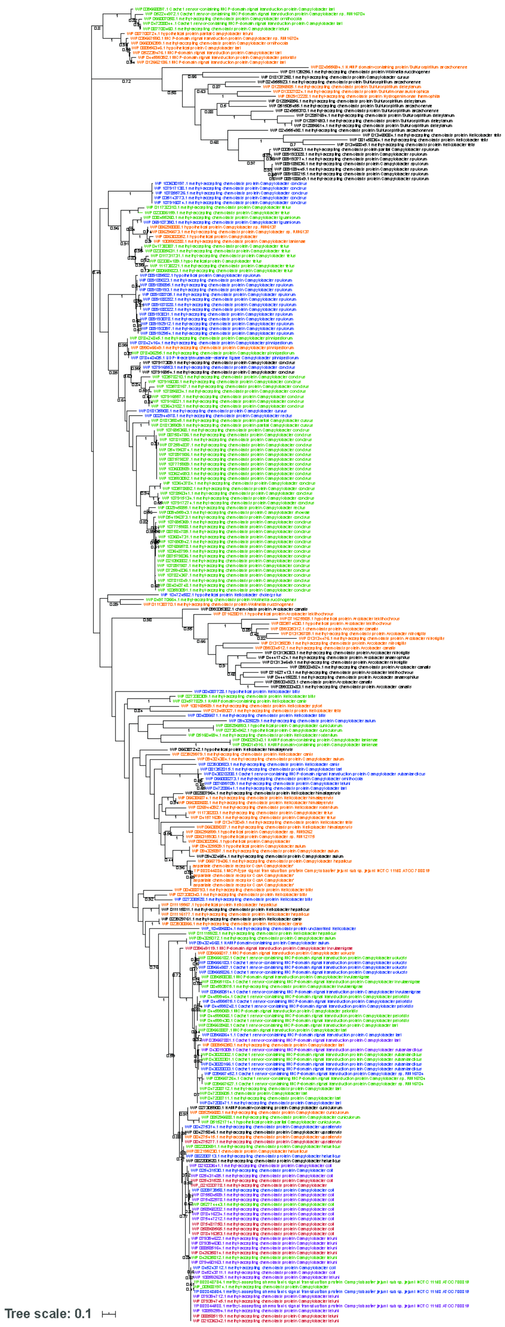

Figure S5. Surface Plasmon Resonance (SPR) sensograms for Tlp3^{peri}, Tlp2^{peri} and Tlp4^{peri}. Single cycle SPR plots are shown as concentration dependent interactions between proteins and monosaccharides as illustrated.

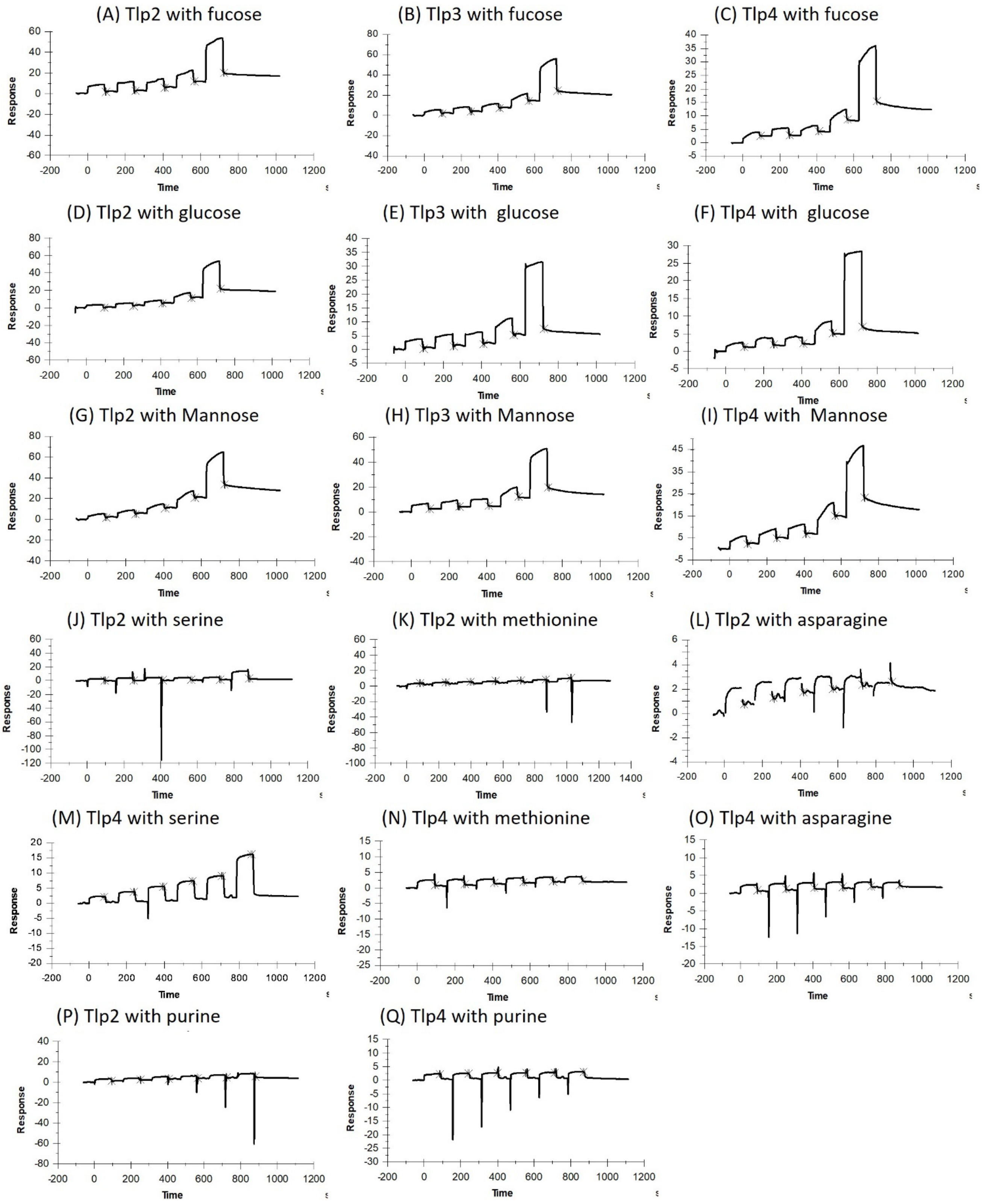

Figure S6. Competition between glucose and fucose /glucose and malic acid binding to Tlp3. **A)** Injections are indicated as injection-A (PBS or glucose at 300 μM for 30 seconds) and injection-B (glucose at 300 μM or fucose at 300 µM for 60 seconds). Final responses units (RU) of injections are recorded (indicated by dashed vertical line and label), with the response of glucose equalling 5.07RU ± 0.81 at 15 seconds after injection-B and l fucose equalling 9.86 RU ± 1.14 at 15 seconds after injection-B. The response 15 seconds after injection-B when glucose and fucose were competed was 4.4 RU ± 0.46. As this value sits below 5 RU, it indicates direct competition for the same site where glucose replacing and blocking the bound fucose during the 60 second injection-B. **B)** A) Injections are indicated as injection-A (PBS or glucose at 500 μM for 30 seconds) and injection-B (glucose at 300 μM or malic acid at 200 µM for 60 seconds). Final responses units (RU) of injections are recorded (indicated by dashed vertical line and label), with the response of glucose equalling 5.07RU ± 0.81 at 15 seconds after injection-B and l malic acid equalling 13.5 RU ± 2.4 at 15 seconds after injection-B. The response 15 seconds after injection-B when glucose and malic acid were competed was 10.04 RU ± 0.66. As this value sits between 5.07-13.5 RU, it indicates direct competition for the same site with glucose replacing some of the bound malic acid during the 60 second injection-B.

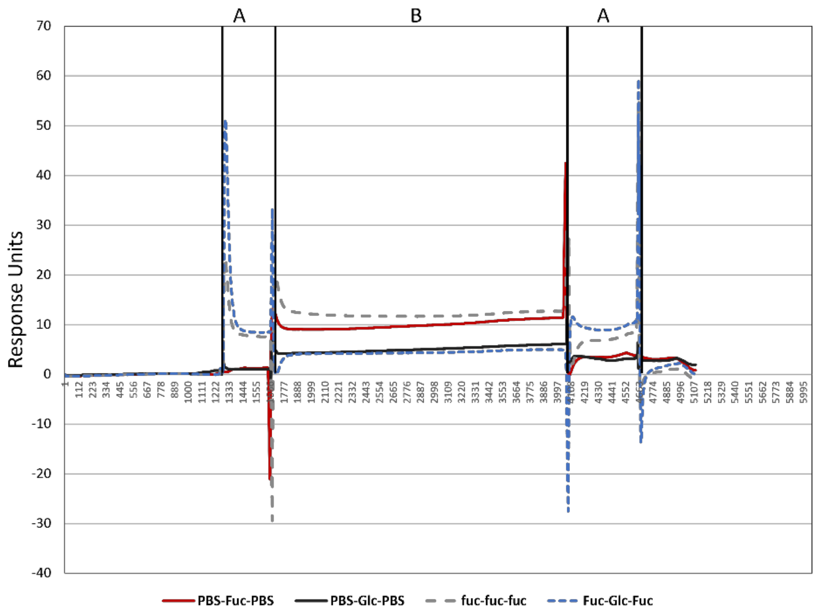
A
B
A
Response Units
PBS-Fuc-PBS
PBS-Glc-PBS
fuc-fuc-fuc
Fuc-Glc-Fuc

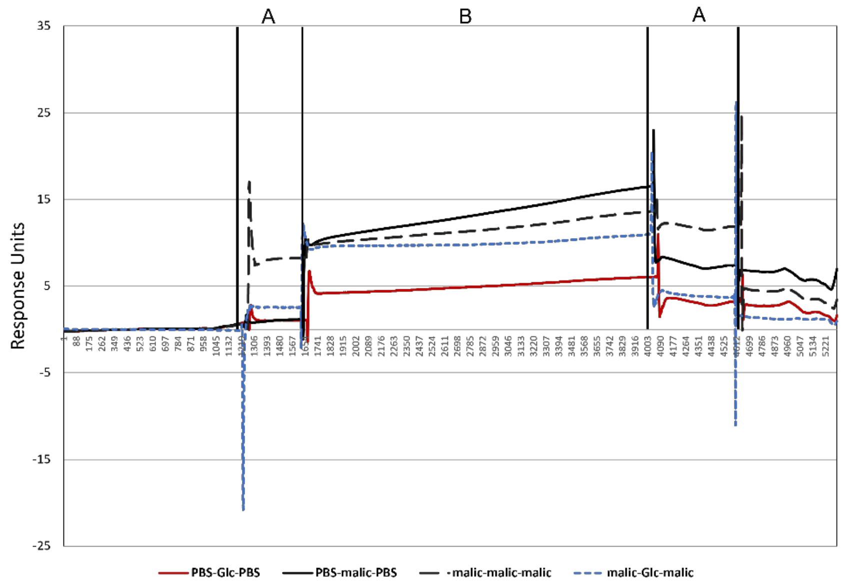
A
B
A
Response Units
PBS-Glc-PBS
PBS-malic-PBS
malic-malic-malic
malic-Glc-malic

Figure S7. SPR ligand competition analysis for Tlp3. SPR competition analysis of binding of glucose (glc), α-ketoglutarate (aketo), malic acid (malic), lysine (lys), glucosamine (glcA), arginine (arg), and isoleucine (iso) to WT Tlp3^{LBD}. The experimental RU value is actual response unit (RU) values for the following: A) (glc) is responses to glucose only, (aketo) is responses to α-ketoglutarate only, and (glc+aketo) is glucose responses following saturation with α-ketoglutarate and (aketo+glc) is α-ketoglutarate following saturation with glucose. B) (glc) is responses to glucose only, (malic) is responses to malic acid only, and (glc+malic) is glucose responses following saturation with malic acid and (malic+glc) is malic acid following saturation with glucose. C) (glc) is responses to glucose only, (lys) is responses to lysine only, and (glc +lys) is glucose responses following saturation with lysine and (lys+glc) is lysine following saturation with glucose. D) (glc) responds to glucose only, (glcA) is response to glucosamine only, (glc+glcA) glucose response following saturation with glucosamine and (glcA+glc) glucosamine following saturation with glucose. E) (iso) is responses to isoleucine only, (arg) is responses to arginine only, and (iso+arg) is isoleucine responses following saturation with arginine and (arg+iso) is arginine following saturation with isoleucine. F) (iso) is responses to isoleucine only, (malic) is responses to malic acid only, and (iso+ malic) is isoleucine responses following saturation with malic acid and (malic +Iso) is malic acid following saturation with isoleucine. G) (iso) is responses to isoleucine only, (lys) is responses to lysine only, and (iso+lys) is isoleucine responses following saturation with lysine and (lys+iso) is lysine following saturation with isoleucine. The theoretical value is responses unites (RU) values based on mathematical theory. The binding status of the ligands to protein are classified as: Independent site (additive/accumulative effect); ligands binding to different binding sites, Shared site; ligands binding/sharing same binding site, Preferential Shared site; ligands binding/sharing same binding site but the protein binds to one ligand better than the other when in equilibrium. All response data was normalized 100 Da molecular weight for each analyte allowing direct comparison of responses.

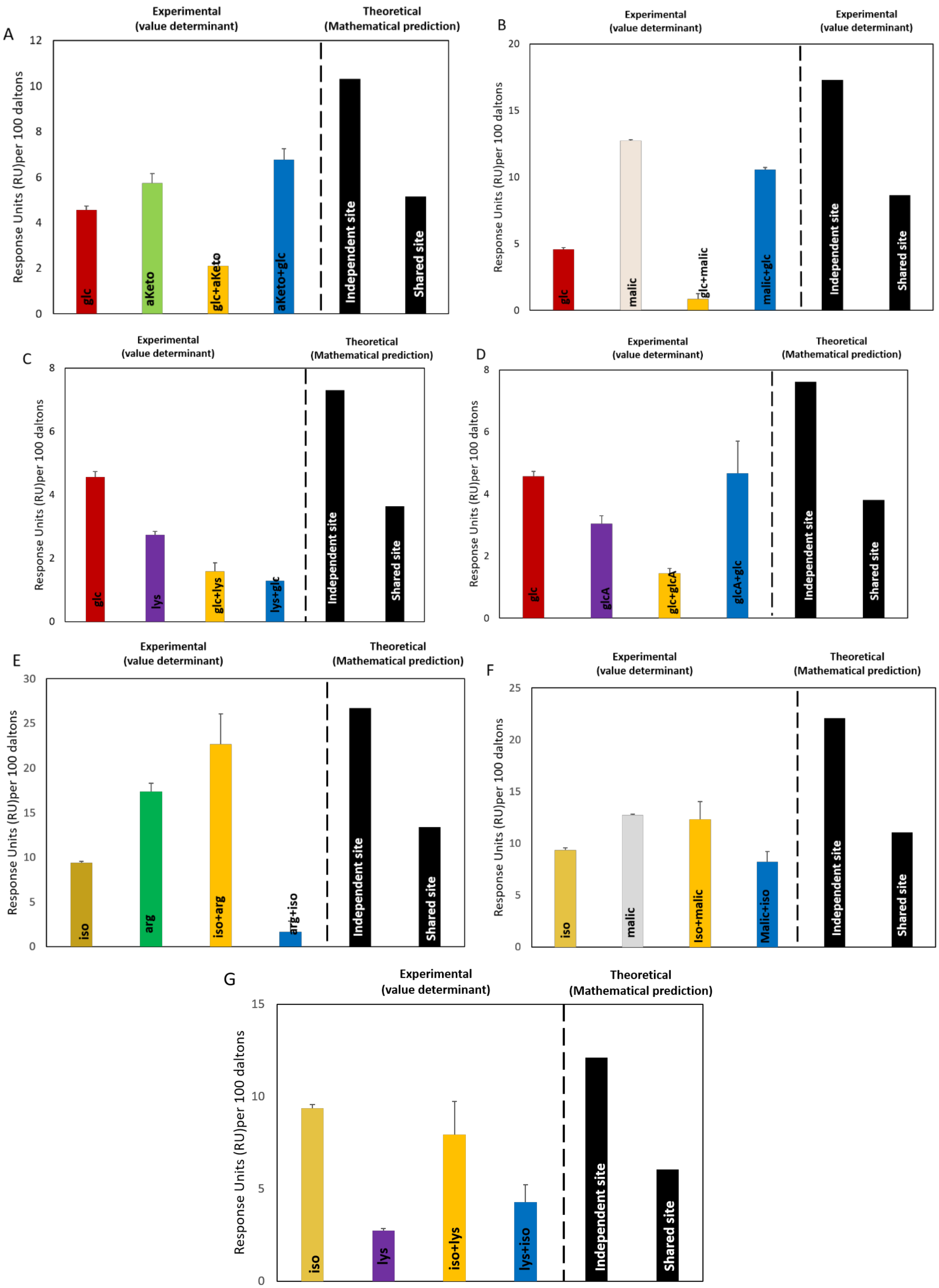
A
Experimental (value determinant)
Theoretical (Mathematical prediction)
Response Units (RU)per 100 daltons
0
2
4
6
8
10
12
glc
aKeto
glc+aKeto
aKeto+glc
Independent site
Shared site
B
Experimental (value determinant)
Experimental (value determinant)
Response Units (RU)per 100 daltons
0
5
10
15
20
glc
malic
glc+malic
malic+glc
Independent site
Shared site
C
Experimental (value determinant)
Theoretical (Mathematical prediction)
Response Units (RU)per 100 daltons
0
2
4
6
8
glc
lys
glc+lys
lys+glc
Independent site
Shared site
D
Experimental (value determinant)
Theoretical (Mathematical prediction)
Response Units (RU)per 100 daltons
0
2
4
6
8
glc
glcA
glc+glcA
glcA+glc
Independent site
Shared site
E
Experimental (value determinant)
Theoretical (Mathematical prediction)
Response Units (RU)per 100 daltons
0
5
10
15
20
25
30
iso
arg
iso+arg
arg+iso
Independent site
Shared site
F
Experimental (value determinant)
Theoretical (Mathematical prediction)
Response Units (RU)per 100 daltons
0
5
10
15
20
25
iso
malic
Iso+malic
Malic+iso
Independent site
Shared site
G
Experimental (value determinant)
Theoretical (Mathematical prediction)
Response Units (RU)per 100 daltons
0
5
10
15
iso
lys
iso+lys
lys+iso
Independent site
Shared site

Figure S8. Modified hard plug agar assay (tHAP) chemotaxis assay quantitation. Viable counts of *C. jejuni* are shown on a log scale. The agarose plugs contained the following ligands: (A) fucose, glucose, galactose, mannose and sialic acid; (B) serine, methionine, asparagine, lysine, cysteine, purine and aspartate. *C. jejuni* 81116 *flaA*$^-$/*flab*$^-$ isogenic mutant was used as a non-motile, non-chemotactic control; and agar plugs containing no added ligand were used as a negative control. Standard errors are shown as bars above the mean of three replicates.

(A)

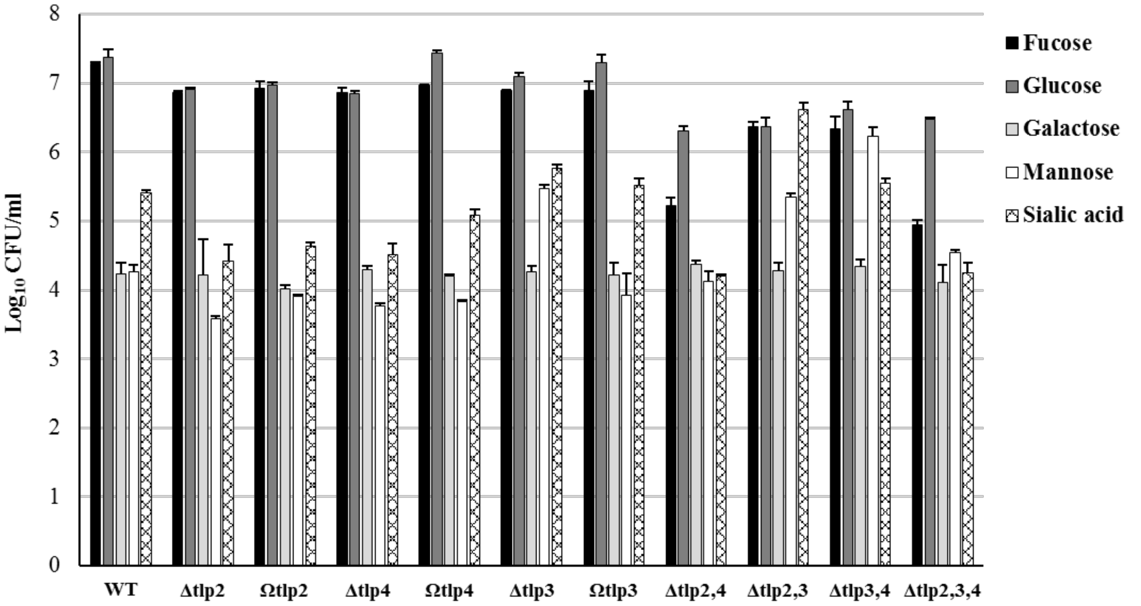

(B)

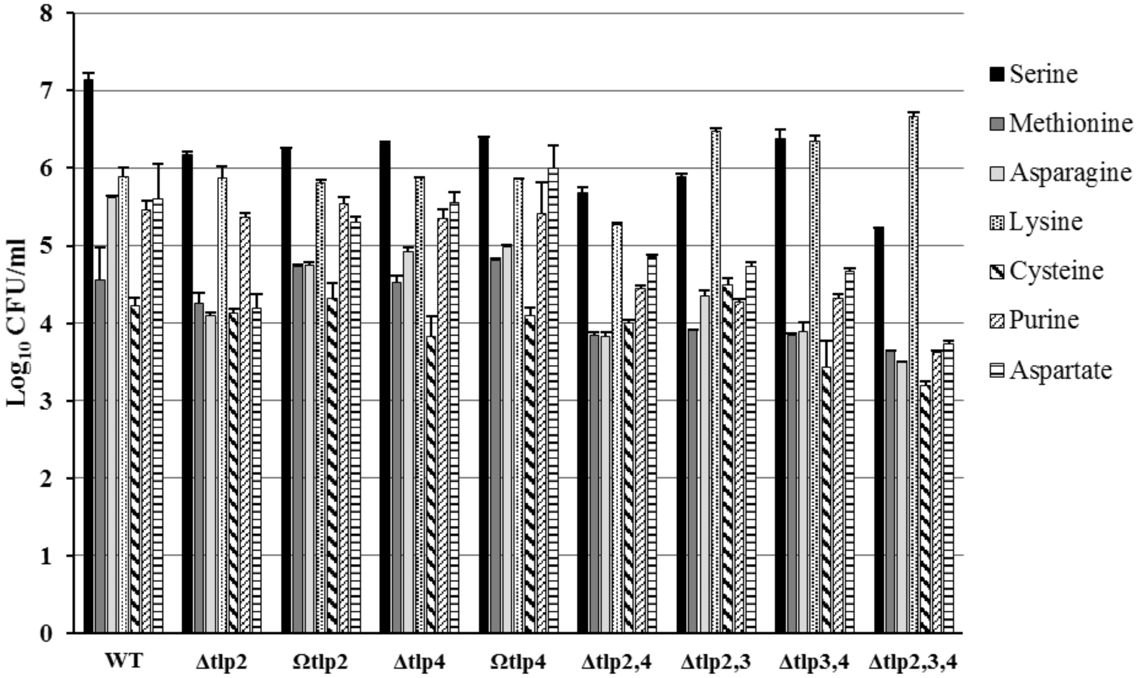

Figure S9. Circular dichroism spectrometry analyses of periplasmic domains of **(A)** Tlp2, **(B)** Tlp3 and **(C)** Tlp4. Molar ellipticity [θ] is calculated using the formula: (θ × 100 × M)/(C × l × n). Purified Tlp2, Tlp3 and Tlp4 were confirmed as folded proteins consisting of α-helix structures.

(A) Tlp2

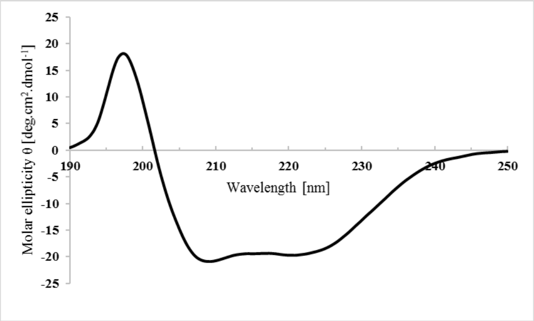

(B) Tlp3

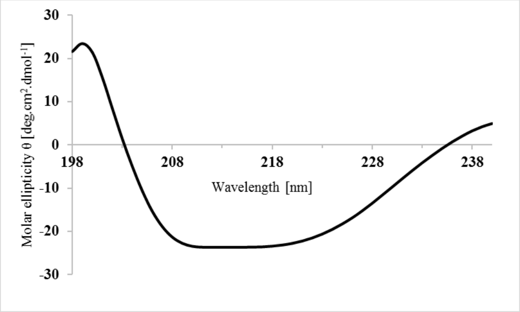

(C) Tlp4

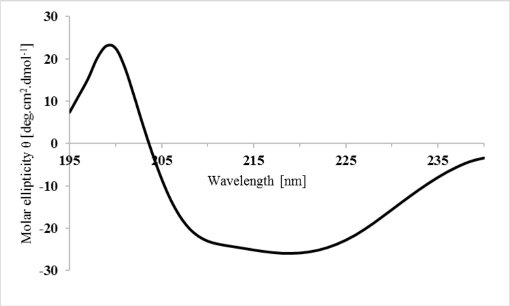

Videos S1. Molecular dynamics simulations between two molecules, a monosaccharide (glucose) and an amino acid (arginine).

Videos S2. Molecular dynamics simulations between two molecules, an amino acid (arginine) and a monosaccharide (glucose).

Table S1. The presence of dCache_1 domain containing chemoreceptors in different *Campylobacterota* representatives.

Phylum	Class	Order	Family	Genus	GTDB Species name	Representative genome	NCBI name	MiST ID	NCBI ID	
**p_Campylobacterota**										
	c_Campylobacteria									
		o_Campylobacteriales								
			f_Arcobacteraceae							
				Aliarcobacter	*Arcobacter butzleri*	GCF_001010585.1	*Arcobacter butzleri L348*			F7 system, 36H
				Arcobacter	*Arcobacter nitrofigilis*	GCF_000092245.1	*Arcobacter nitrofigilis DSM 7299*	GCF_000092245.1-ARNIT_RS12945	WP_013136383.1	F7 system, **36H**
								GCF_000092245.1-ARNIT_RS10155	WP_013135839.1	
								GCF_000092245.1-ARNIT_RS04225	WP_013134649.1	
								GCF_000092245.1-ARNIT_RS15045	WP_013136785.1	
								GCF_000092245.1-ARNIT_RS03330	WP_013134476.1	
				Halarcobacter	*Halarcobacter anaerophilus*	GCF_000935065.1	*Arcobacter anaerophilus*	GCF_000935065.1-TN41_RS08535	WP_044417424.1	F7 system, **36H**
								GCF_000935065.1-TN41_RS03965	WP_044415828.1	
				Malaciobacter	*Malaciobacter canalis*	GCF_002723485.1	*Arcobacter canalis*	GCF_002723485.1-CPG37_RS07870	WP_099334512.1	F7 system, F9 system, 34H, **36H**, 40H, 44H
								GCF_002723485.1-CPG37_RS13415	WP_099335382.1	
								GCF_002723485.1-CPG37_RS01535	WP_099333483.1	
								GCF_002723485.1-CPG37_RS10520	WP_099334924.1	
								GCF_002723485.1-CPG37_RS10515	WP_099334923.1	
								GCF_002723485.1-CPG37_RS12925	WP_099335312.1	
				Poseidonibacter	*Poseidonibacter lekithochrous*	GCF_001878855.1	*Arcobacter lekithochrous*	GCF_001878855.1-BRO21_RS12110	WP_071627413.1	F7 system, F8 system, 34H, **36H**
								GCF_001878855.1-BRO21_RS15155	WP_071628011.1	
								GCF_001878855.1-BRO21_RS08205	WP_083574630.1	
								GCF_001878855.1-BRO21_RS05035	WP_071625985.1	

f_Campylobacteraceae							
	Campylobacter						
		Campylobacter fetus	GCF_900475935.1	*Campylobacter fetus*	GCF_900475935.1-DQN38_RS08290	WP_011732310.1	F3 system; 24H, **28H**, 40H, 44H
					GCF_900475935.1-DQN38_RS06385	WP_041738387.1	
					GCF_900475935.1-DQN38_RS06380	WP_111738221.1	
					GCF_900475935.1-DQN38_RS00960	WP_011731731.1	
		Campylobacter iguaniorum	GCF_000736415.1	*Campylobacter iguaniorum*	GCF_000736415.1-CIG1485E_RS09305	WP_038455260.1	F3 system; **28H**, 40H
					GCF_000736415.1-CIG1485E_RS09275	WP_069107390.1	
		Campylobacter lanienae	GCF_002139415.1	*Campylobacter lanienae*	GCF_002139415.1-CCB79_RS06750	WP_096025340.1	F3 system; **28H**, 40H, 44H
		Campylobacter sp002139855	GCF_002139855.1	*Campylobacter sp. RM6137*	GCF_002139855.1-CSUIS_RS06325	WP_086298008.1	F3 system; **28H**, 40H
					GCF_002139855.1-CSUIS_RS00300	WP_086296673.1	
		Campylobacter sp002139875	GCF_002139875.1	*Campylobacter sp. RM12175*	GCF_002139875.1-CVIC12175_RS06330	WP_086315930.1	F3 system; **28H**, 40H, 44H
			GCF_002139175.1	*Campylobacter sp. RM9262*	GCF_002139175.1-CVIC9262_RS00905	WP_086256599.1	F3 system; **28H**, 40H, 44H
		Campylobacter sp002139915	GCF_002139915.1	*Campylobacter sp. NCTC 13003*	GCF_002139915.1-CIGN_RS01655	WP_086302052.1	F3 system; **28H**, 40H
					GCF_002139915.1-CIGN_RS01725	WP_086302056.1	
	Campylobacter_A						
		Campylobacter_A concisus	GCF_001298465.1	*Campylobacter concisus*	GCF_001298465.1-CCON33237_RS03260	WP_054196374.1	F3 system, **28H**, 40H
					GCF_001298465.1-CCON33237_RS03255	WP_054196373.1	
		Campylobacter_A concisus_N	GCF_002914005.1	*Campylobacter concisus*	GCF_002914005.1-CYP54_RS07185	WP_103600989.1	F3 system, **28H**, 40H
					GCF_002914005.1-CYP54_RS07180	WP_103604731.1	
		Campylobacter_A concisus_U	GCF_003048575.1	*Campylobacter concisus*	GCF_003048575.1-CVS84_RS01985	WP_107690942.1	F3 system, **28H**, 40H

		Campylobacter_A pinnipediorum	GCF_002021925.1	*Campylobacter pinnipediorum subsp. Pinnipediorum*	GCF_002021925.1-CPIN17260_RS08940	WP_078406295.1	F3 system, **28H**, 40H
					GCF_002021925.1-CPIN17260_RS00765	WP_099046649.1	
					GCF_002021925.1-CPIN17260_RS00760	WP_078440405.1	
		Campylobacter_A rectus	GCF_000174175.1	*Campylobacter rectus RM3267*	GCF_000174175.1-CAMRE0001_RS08300	WP_002944978.1	F3 system, **28H**, 40H
					GCF_000174175.1-CAMRE0001_RS11105	WP_002945855.1	
		Campylobacter_A showae_A	GCF_000313615.1	*Campylobacter showae CSUNSWCD*	GCF_000313615.1-CSUNSWCD_RS08695	WP_009495943.1	F3 system, **28H**, 40H
	Campylobacter_B						
		Campylobacter_B sputorum	GCF_002220755.1	*Campylobacter sputorum bv. paraureolyticus*	GCF_002220755.1-CSPARA_RS08305	WP_089189895.1	F3 system, **28H**, **40H**, 44H
					GCF_002220755.1-CSPARA_RS05480	WP_089189193.1	
					GCF_002220755.1-CSPARA_RS01695	WP_089189023.1	
					GCF_002220755.1-CSPARA_RS05130	WP_089189502.1	
					GCF_002220755.1-CSPARA_RS07910	WP_089189836.1	40H
					GCF_002220755.1-CSPARA_RS04765	WP_089189445.1	40H
	Campylobacter_D						
		Campylobacter_D avium	GCF_002238335.1	*Campylobacter avium LMG 24591*	GCF_002238335.1-CAV_RS08355	WP_094324384.1	F3 system, **28H**, 40H, 44H
					GCF_002238335.1-CAV_RS07060	WP_094325829.1	
					GCF_002238335.1-CAV_RS01035	WP_094324668.1	
					GCF_002238335.1-CAV_RS00955	WP_094324654.1	
					GCF_002238335.1-CAV_RS07455	WP_094325897.1	
					GCF_002238335.1-CAV_RS00870	WP_094325509.1	

		GCF_002245 935.1	*Campylobac ter avium*	GCF_002245935.1-CAV8706_RS07895	WP_094325 829.1	F3 system, **28H**, 40H, 44H
				GCF_002245935.1-CAV8706_RS01820	WP_094324 654.1	
				GCF_002245935.1-CAV8706_RS04075	WP_094325 072.1	
				GCF_002245935.1-CAV8706_RS01900	WP_094324 668.1	
				GCF_002245935.1-CAV8706_RS00375	WP_094324 384.1	
				GCF_002245935.1-CAV8706_RS01735	WP_094325 509.1	
				GCF_002245935.1-CAV8706_RS08285	WP_094325 897.1	
	Campylobact er_D cuniculorum	GCF_002104 335.1	*RS Campylobac ter cuniculorum DSM 23162 = LMG 24588*	GCF_002104335.1-CCUN_RS08200	WP_085296 680.1	F3 system, **28H**, 40H
				GCF_002104335.1-CCUN_RS06815	WP_027305 900.1	
				GCF_002104335.1-CCUN_RS09175	WP_085296 688.1	
				GCF_002104335.1-CCUN_RS00300	WP_085296 593.1	
		GCF_000621 005.1	*Campylobac ter cuniculorum DSM 23162 = LMG 24588*	GCF_000621005.1-Q330_RS0106010	WP_027305 900.1	F3 system, **28H**, 40H
				GCF_000621005.1-Q330_RS0105540	WP_051521 714.1	
				GCF_000621005.1-Q330_RS0100570	WP_027304 962.1	
	Campylobact er_D helveticus	GCF_002080 395.1	*Campylobac ter helveticus ATCC 51209*	GCF_002080395.1-CHELV3228_RS00050	WP_082200 713.1	F3 system, **28H**, 40H, 44H
				GCF_002080395.1-CHELV3228_RS09175	WP_082200 691.1	
				GCF_002080395.1-CHELV3228_RS01550	WP_082199 230.1	
				GCF_002080395.1-CHELV3228_RS08180	WP_082200 520.1	

	Campylobacter_D upsaliensis	GCF_000167395.1	*Campylobacter upsaliensis RM3195*	GCF_000167395.1-CUP_RS03190	WP_004275314.1	F3 system, **28H**, 40H, 44H
				GCF_000167395.1-CUP_RS03010	WP_004275277.1	
				GCF_000167395.1-CUP_RS03665	WP_004275415.1	
				GCF_000167395.1-CUP_RS05805	WP_004275846.1	
	Campylobacter_D sp002738235	GCF_002738235.1	*Campylobacter sp. 73/13*			F3 system, 40H, 44H
	Campylobacter_D hepaticus	GCF_001687475.1	*Campylobacter hepaticus*	GCF_001687475.1-A2J15_RS07055	WP_066779406.1	F3 system, **28H**, 40H
	Campylobacter_D jejuni_B	GCF_002179165.1	*Campylobacter jejuni W1*			F3 system, 28H, 40H, 44H
	Campylobacter_D coli	GCF_000465235.1	*Campylobacter coli CVM N29710*	GCF_000465235.1-G157_RS01370	WP_002822567.1	F3 system, **28H**, 40H
				GCF_000465235.1-G157_RS01095	WP_021033641.1	
				GCF_000465235.1-G157_RS07430	WP_021033778.1	
		GCF_000465235.1	*Campylobacter coli CVM N29710*	GCF_000465235.1-G157_RS01370	WP_002822567.1	F3 system, **28H**, 40H
				GCF_000465235.1-G157_RS01095	WP_021033641.1	
				GCF_000465235.1-G157_RS07430	WP_021033778.1	
		GCF_000583795.1	*Campylobacter coli RM5611*	GCF_000583795.1-YSU_RS08010	WP_025431530.1	F3 system, **28H**, 40H
				GCF_000583795.1-YSU_RS01055	WP_025431485.1	
				GCF_000583795.1-YSU_RS07490	WP_025431528.1	
		GCF_000954195.1	*Campylobacter coli*	GCF_000954195.1-VC76_RS07390	WP_002822567.1	F3 system, **28H**, 40H
				GCF_000954195.1-VC76_RS07950	WP_045243712.1	
				GCF_000954195.1-VC76_RS07680	WP_045243711.1	
				GCF_000954195.1-VC76_RS01340	WP_021033778.1	

	Campylobacter_D coli_A	GCF_000470055.1	*Campylobacter coli 76339*	GCF_000470055.1-BN865_RS01275	WP_020973958.1	F3 system, **28H**, 40H
		GCF_001498475.1	*Campylobacter coli*	GCF_001498475.1-AV450_RS02685	WP_075504589.1	F3 system, **28H**, 40H
		GCF_001494895.1	*Campylobacter coli*	GCF_001494895.1-AV288_RS04180	WP_075482578.1	F3 system, **28H**, 40H
	Campylobacter_D coli_B	GCF_001492295.1	*Campylobacter coli*	GCF_001492295.1-AV154_RS00980	WP_058968202.1	F3 system, **28H**, 40H
				GCF_001492295.1-AV154_RS08260	WP_058969565.1	
		GCF_002019865.1	*Campylobacter coli*	GCF_002019865.1-BOQ00_RS06585	WP_078416234.1	F3 system, **28H**, 40H
				GCF_002019865.1-BOQ00_RS08225	WP_078416353.1	
		GCF_002020015.1	*Campylobacter coli VA8*	GCF_002020015.1-BOP99_RS05705	WP_075447212.1	F3 system, **28H**, 40H
				GCF_002020015.1-BOP99_RS05315	WP_052774443.1	
				GCF_002020015.1-BOP99_RS00005	WP_075481750.1	
	Campylobacter_D jejuni	**GCF_000009085.1**	***Campylobacter jejuni subsp. jejuni NCTC 11168 = ATCC 700819***	GCF_000009085.1-Cj1506c	**YP_002344885.1**	F3 system, **28H**, 40H
				GCF_000009085.1-Cj0144	**YP_002343604.1**	
				GCF_000009085.1-Cj1564	**YP_002344933.1**	
				GCF_000009085.1-Cj0262c	**YP_002343704.1**	
		GCF_000835285.1	*Campylobacter jejuni subsp. jejuni*	GCF_000835285.1-PJ17_RS07875	WP_002858883.1	F3 system, **28H**, 40H
				GCF_000835285.1-PJ17_RS00730	WP_079394622.1	
				GCF_000835285.1-PJ17_RS08150	WP_079394630.1	
				GCF_000835285.1-PJ17_RS01290	WP_042635812.1	
				GCF_000835285.1-PJ17_RS01300	WP_042635814.1	
		GCF_002209045.1	*Campylobacter jejuni subsp. jejuni*	GCF_002209045.1-A6J93_RS07640	WP_002871853.1	F3 system, **28H**, 40H
				GCF_002209045.1-A6J93_RS00985	WP_079460163.1	

				GCF_002209045.1-A6J93_RS07895	WP_088585164.1	
				GCF_002209045.1-A6J93_RS01525	WP_088585119.1	
		GCF_003060745.1	*Campylobacter jejuni*	GCF_003060745.1-CJ12660_RS07665	WP_002851362.1	F3 system, **28H**, 40H
				GCF_003060745.1-CJ12660_RS07910	WP_108592625.1	
				GCF_003060745.1-CJ12660_RS00720	WP_108592594.1	
				GCF_003060745.1-CJ12660_RS01265	WP_021036342.1	
		GCF_000835345.1	*Campylobacter jejuni subsp. jejuni*	GCF_000835345.1-PJ19_RS08250	WP_002851362.1	F3 system, **28H**, 40H
				GCF_000835345.1-PJ19_RS00730	WP_079394712.1	
				GCF_000835345.1-PJ19_RS08495	WP_079394745.1	
				GCF_000835345.1-PJ19_RS01250	WP_009881974.1	
	Campylobacter_D insulaenigrae	GCF_000816185.1	*Campylobacter insulaenigrae NCTC 12927*	GCF_000816185.1-CINS_RS06385	WP_039650838.1	F3 system, **28H**, 40H, 44H
				GCF_000816185.1-CINS_RS07375	WP_039651184.1	
				GCF_000816185.1-CINS_RS00645	WP_084593975.1	
				GCF_000816185.1-CINS_RS05400	WP_039650514.1	
				GCF_000816185.1-CINS_RS01075	WP_039649119.1	
	Campylobacter_D volucris	GCF_000816345.1	*Campylobacter volucris LMG 24379*	GCF_000816345.1-CVOL_RS07670	WP_039666183.1	F3 system, **28H**, 40H, 44H
				GCF_000816345.1-CVOL_RS07665	WP_039666182.1	
				GCF_000816345.1-CVOL_RS06585	WP_039666017.1	
				GCF_000816345.1-CVOL_RS05505	WP_039665826.1	
				GCF_000816345.1-CVOL_RS00675	WP_039664987.1	
	Campylobacter_D peloridis	GCF_000816785.1	*Campylobacter peloridis LMG 23910*	GCF_000816785.1-CPEL_RS08275	WP_044599430.1	F3 system, **28H**, 40H
				GCF_000816785.1-CPEL_RS07205	WP_044599248.1	

				GCF_000816785.1-CPEL_RS06215	WP_044599069.1	
				GCF_000816785.1-CPEL_RS06210	WP_044599575.1	
				GCF_000816785.1-CPEL_RS06205	WP_044599068.1	
				GCF_000816785.1-CPEL_RS00725	WP_044599464.1	
				GCF_000816785.1-CPEL_RS08030	WP_044599392.1	
	Campylobacter_D sp000816245	GCF_000816245.1	*Campylobacter sp. RM16704*	GCF_000816245.1-CAQ16704_RS07750	WP_039667627.1	F3 system, **28H**, 40H, 44H
				GCF_000816245.1-CAQ16704_RS06675	WP_039667452.1	
				GCF_000816245.1-CAQ16704_RS05595	WP_039667264.1	
				GCF_000816245.1-CAQ16704_RS02125	WP_052244972.1	
				GCF_000816245.1-CAQ16704_RS07495	WP_039667590.1	
	Campylobacter_D subantarcticus	GCF_000816305.1	*Campylobacter subantarcticus LMG 24377*	GCF_000816305.1-CSUB8523_RS09320	WP_043020302.1	F3 system, **28H**, 40H
				GCF_000816305.1-CSUB8523_RS09315	WP_043020301.1	
				GCF_000816305.1-CSUB8523_RS08420	WP_043020208.1	
				GCF_000816305.1-CSUB8523_RS08075	WP_043020166.1	
				GCF_000816305.1-CSUB8523_RS07005	WP_043020033.1	
				GCF_000816305.1-CSUB8523_RS00730	WP_043019309.1	
	Campylobacter_D ornithocola	GCF_001705345.1	*Campylobacter ornithocola WBE38*	GCF_001705345.1-A7X81_RS05805	WP_066008273.1	F3 system, **28H**, 40H, 44H
				GCF_001705345.1-A7X81_RS02290	WP_066007058.1	
				GCF_001705345.1-A7X81_RS00225	WP_066006399.1	
	Campylobacter_D lari	GCF_900111465.1	*Campylobacter lari subsp. Lari ATCC 35221*	GCF_900111465.1-BMY58_RS03220	WP_081352015.1	F3 system, **28H**, 40H, 44H
				GCF_900111465.1-BMY58_RS06285	WP_012662186.1	

		Campylobacter_D lari_B	GCF_000816225.1	*Campylobacter lari subsp. concheus LMG 11760*	GCF_000816225.1-CONCH_RS07460	WP_039668968.1	F3 system, **28H**, 40H, 44H
					GCF_000816225.1-CONCH_RS06405	WP_039668807.1	
					GCF_000816225.1-CONCH_RS00695	WP_039667881.1	
					GCF_000816225.1-CONCH_RS05400	WP_039668641.1	
					GCF_000816225.1-CONCH_RS02065	WP_039668097.1	
					GCF_000816225.1-CONCH_RS07215	WP_052239476.1	
		Campylobacter_D lari_C	GCF_001017575.1	*Campylobacter lari*	GCF_001017575.1-CD56_RS06815	WP_047208605.1	F3 system, **28H**, 40H, 44H
					GCF_001017575.1-CD56_RS05810	WP_047208471.1	
					GCF_001017575.1-CD56_RS07085	WP_047208641.1	
					GCF_001017575.1-CD56_RS07865	WP_047208712.1	
					GCF_001017575.1-CD56_RS07860	WP_047208711.1	
					GCF_001017575.1-CD56_RS02030	WP_047208044.1	
					GCF_001017575.1-CD56_RS08150	WP_080956350.1	
					GCF_001017575.1-CD56_RS07615	WP_080956346.1	
		Campylobacter_D jejuni_C	GCF_002179635.1	*Campylobacter jejuni S4*	GCF_002179635.1-CAS13_RS02695	WP_087699709.1	F3 system, **28H**, 40H, 44H
					GCF_002179635.1-CAS13_RS06905	WP_087700724.1	
					GCF_002179635.1-CAS13_RS05225	WP_087700460.1	
f_Helicobacteraceae							
	Helicobacter	*Helicobacter pylori*	GCF_900478295.1	*Helicobacter pylori NCTC 11637 = CCUG 17874 = ATCC 43504*	GCF_900478295.1-DQL14_RS01445	WP_108169589.1	F3 system, **28H**, 40H
	Helicobacter_A	*Helicobacter_A bilis*	GCF_000686565.1	*Helicobacter bilis ATCC 51630*	GCF_000686565.1-T368_RS13380	WP_004087959.1	F3 system, **28H**, 40H, 44H

					GCF_000686565.1-T368_RS0105195	WP_027338340.1	
					GCF_000686565.1-T368_RS0103980	WP_027338309.1	
					GCF_000686565.1-T368_RS0112100	WP_027338528.1	
					GCF_000686565.1-T368_RS0111695	WP_004086671.1	
					GCF_000686565.1-T368_RS0101325	WP_004088793.1	
					GCF_000686565.1-T368_RS0104865	WP_004087728.1	
	Helicobacter_B	*Helicobacter_B canis*	GCF_000507865.1	*Helicobacter canis NCTC 12740*	GCF_000507865.1-HMPREF2087_RS09205	WP_034577829.1	F3 system, **28H**, 40H, 44H
					GCF_000507865.1-HMPREF2087_RS07780	WP_023930583.1	
					GCF_000507865.1-HMPREF2087_RS04115	WP_023929761.1	
					GCF_000507865.1-HMPREF2087_RS09050	WP_023930866.1	
					GCF_000507865.1-HMPREF2087_RS05180	WP_023929979.1	
	Helicobacter_C	*Helicobacter_C hepaticus*	GCF_000007905.1	*Helicobacter hepaticus ATCC 51449*	GCF_000007905.1-HH_RS05325	WP_011115928.1	F3 system, **28H**, 40H, 44H
					GCF_000007905.1-HH_RS04770	WP_011115811.1	
					GCF_000007905.1-HH_RS06465	WP_011116177.1	
					GCF_000007905.1-HH_RS03595	WP_011115567.1	
	Helicobacter_D	*Helicobacter_D rodentium*	GCF_000687535.1	*Helicobacter rodentium ATCC 700285*	GCF_000687535.1-BS85_RS10280	WP_051604694.1	F3 system, **28H**, 40H, 44H
					GCF_000687535.1-BS85_RS0106525	WP_026944092.1	
	Helicobacter_E	*Helicobacter_E felis*	GCF_000200595.1	*Helicobacter felis ATCC 49179*	GCF_000200595.1-HFELIS_RS04385	WP_013469327.1	F3 system, 24H, **28H**, **40H,** 44H
					GCF_000200595.1-HFELIS_RS07975	WP_013470049.1	
					GCF_000200595.1-HFELIS_RS02830	WP_081458364.1	40H
					GCF_000200595.1-HFELIS_RS03120	WP_013469084.1	40H
					GCF_000200595.1-HFELIS_RS01885	WP_013468845.1	40H

		Helicobacter_F	*Helicobacter_F himalayensis*	GCF_001602095.1	*Helicobacter himalayensis*	GCF_001602095.1-A3217_RS04305	WP_082807964.1	F3 system, **28H**, 40H, 44H
						GCF_001602095.1-A3217_RS01860	WP_066387242.1	
						GCF_001602095.1-A3217_RS06775	WP_066389087.1	
						GCF_001602095.1-A3217_RS00950	WP_066386874.1	
						GCF_001602095.1-A3217_RS05205	WP_066388688.1	
		Helicobacter_G	*Helicobacter_G cholecystus*	GCF_900198475.1	*Helicobacter cholecystus*	GCF_900198475.1-C6H20_RS03470	WP_104724582.1	F3 system, 24H, **28H**
		Helicobacter_H	*Helicobacter_H sp900199585*	GCF_900199585.1	*Helicobacter sp. 'house sparrow 1'*	GCF_900199585.1-C6H31_RS00220	WP_104696804.1	F3 system, 24H, **28H,** 44H
		Helicobacter_I	*Helicobacter_I sp002272925*	GCF_002272925.1	*Helicobacter sp. 13S00401-1*			F3 system, 24H, 28H, 44H
		Helicobacter_J	*Helicobacter_J sp002272795*	GCF_002272795.1	*Helicobacter sp. 13S00482-2*			F3 system, 24H, 40H, no 28H
		Wolinella	*Wolinella succinogenes*	GCF_000196135.1	*Wolinella succinogenes DSM 1740*	GCF_000196135.1-WS_RS06375	WP_049770664.1	F3 system, **28H,** 36H, **40H**, 44H
						GCF_000196135.1-WS_RS04140	WP_011138770.1	
						GCF_000196135.1-WS_RS06705	WP_011139256.1	40H
	f_Hydrogenimonadaceae							
		Hydrogenimonas	*Hydrogenimonas thermophila*	GCF_900115615.1	*Hydrogenimonas thermophila*	GCF_900115615.1-BM227_RS06280	WP_092912228.1	F3, F7, and F8 systems, 28H, 34H, 36H, **40H**, 44H
	f_Nitratiruptoraceae							
		Nitratiruptor	*Nitratiruptor tergarcus*	GCF_900176045.1	*Nitratiruptor tergarcus DSM 16512*			F14, 40H, 44H
	f_Sufurimonadaceae							
		Sulfuricurvum	*Sulfuricurvum kujiense*	GCF_000183725.1	*Sulfuricurvum kujiense DSM 16994*			F3, F7, and F8 systems, 28H, 34H, 36H, **40H**, 44H
		Sulfurimonas	*Sulfurimonas autotrophica*	GCF_000147355.1	*Sulfurimonas autotrophica DSM 16294*	GCF_000147355.1-SAUT_RS06215	WP_013327024.1	F3 and F8 systems, 34H, **40H**

		f_Sulfurospirillaceae							
			Sulfurospirillum	*Sulfurospirillum deleyianum*	GCF_000024885.1	*Sulfurospirillum deleyianum DSM 6946*	GCF_000024885.1-SDEL_RS09760	WP_012857693.1	F3 and F7 systems, 36H, **40H**, 44H
							GCF_000024885.1-SDEL_RS06120	WP_012856985.1	
							GCF_000024885.1-SDEL_RS04195	WP_012856614.1	
							GCF_000024885.1-SDEL_RS09765	WP_012857694.1	
							GCF_000024885.1-SDEL_RS05480	WP_012856856.1	
			Sulfurospirillum_A	*Sulfurospirillum_A arcachonense*	GCF_000597725.1	*Sulfurospirillum arcachonense DSM 9755*	GCF_000597725.1-K354_RS0113080	WP_051505455.1	F3 and F7 systems, 24H, 28H, **36H**, **40H**, 44H
							GCF_000597725.1-K354_RS0110060	WP_024955370.1	40H
							GCF_000597725.1-K354_RS0111745	WP_024955694.1	36H
							GCF_000597725.1-K354_RS14850	WP_024955923.1	40H
							GCF_000597725.1-K354_RS14235	WP_024955498.1	40H
		f_Sulfurovaceae							
			Nitratifractor	*Nitratifractor salsuginis*	GCF_000186245.1	*Nitratifractor salsuginis DSM 16511*			no chemotaxis
			Sufurovum	*Sulfurovum lithotrophicum*	GCF_000987835.1	*Sulfurovum lithotrophicum*			no chemotaxis
	o_Nautiliales								
		f_Nautilliaceae							
			Caminibacter	*Caminibacter mediatlanticus*	GCF_000170735.1	*Caminibacter mediatlanticus TB-2*			F3 system, 28H, 40H, 44H
			Lebetimonas	*Lebetimonas natsushimae*	GCF_002335445.1	*Lebetimonas natsushimae*			F3 system, 40H, 44H
			Nautilia	*Nautilia profundicola*	GCF_000021725.1	*Nautilia profundicola AmH*			F3 system, 28H, 40H, 44H
c_Desulfurellia									
	o_Desulfurellales								
		f_Desulfurellaceae							

		Desulfurella	*Desulfurella acetivorans*	GCF_000517565.1	*Desulfurella acetivorans A63*	F1 system, 40H, 44H
		Desulfurella	*Desulfurella amilsii*	GCF_002119425.1	*Desulfurella amilsii*	F1 system, 40H, 44H
	f_Hippeaceae					
		Hippea	*Hippea maritima*	GCF_000194135.1	*Hippea maritima DSM 10411*	F3 system, 24H, 40H, 44H
		Hippea_A	*Hippea jasoniae*	GCF_000744435.1	*Hippea_A jasoniae*	F3 system, 40H, 44H

Table S2. The presence of tlp2, tlp3 and tlp4 genes in *Campylobacter_D* species with complete and draft genomes.

GTDB Species	Assembly ID	Strain	Tlp2	Tlp3 paralog 1 YNxxxRxW Y x Y x D	Tlp3/CcmL	Tlp3 paralog 2	Tlp4
Campylobacter_D jejuni	GCF_001507125.1	Campylobacter jejuni CJ677CC024					
	GCF_001506885.1	Campylobacter jejuni CJ677CC040					
	GCF_001506205.1	Campylobacter jejuni CJ677CC002					
	GCF_001506345.1	Campylobacter jejuni CJ677CC036					
	GCF_001506465.1	Campylobacter jejuni CJ677CC530					
	GCF_001506665.1	Campylobacter jejuni CJ677CC528					
	GCF_001507145.1	Campylobacter jejuni CJ677CC064					
	GCF_001507065.1	Campylobacter jejuni CJ677CC094			WP_059119483.1		
	GCF_001506485.1	Campylobacter jejuni CJ677CC532					
	GCF_001506705.1	Campylobacter jejuni CJ677CC520					

GCF_001507045.1	Campylobacter jejuni CJ677CC522	
GCF_001506225.1	Campylobacter jejuni CJ677CC534	
GCF_001506445.1	Campylobacter jejuni CJ677CC092	
GCF_001506685.1	Campylobacter jejuni CJ677CC538	
GCF_001506385.1	Campylobacter jejuni CJ677CC016	
GCF_001506425.1	Campylobacter jejuni CJ677CC535	
GCF_001506505.1	Campylobacter jejuni CJ677CC529	
GCF_001506525.1	Campylobacter jejuni CJ677CC531	
GCF_001506565.1	Campylobacter jejuni CJ677CC059	
GCF_001506625.1	Campylobacter jejuni CJ677CC537	WP_059116327.1
GCF_001506745.1	Campylobacter jejuni CJ677CC039	WP_079756629.1
GCF_001506825.1	Campylobacter jejuni CJ677CC078	
GCF_001506905.1	Campylobacter jejuni CJ677CC061	
GCF_001506965.1	Campylobacter jejuni CJ677CC047	WP_079756887.1
GCF_001506985.1	Campylobacter jejuni CJ677CC058	
GCF_001507085.1	Campylobacter jejuni CJ677CC008	
GCF_001506185.1	Campylobacter jejuni CJ677CC519	
GCF_001506545.1	Campylobacter jejuni CJ677CC062	
GCF_001506805.1	Campylobacter jejuni CJ677CC527	
GCF_001507005.1	Campylobacter jejuni CJ677CC013	

GCF_001507025.1	Campylobacter jejuni CJ677CC100	
GCF_001507185.1	Campylobacter jejuni CJ677CC026	
GCF_001506305.1	Campylobacter jejuni CJ677CC526	
GCF_001506365.1	Campylobacter jejuni CJ677CC524	
GCF_001507165.1	Campylobacter jejuni CJ677CC525	
GCF_001506245.1	Campylobacter jejuni CJ677CC536	
GCF_001506285.1	Campylobacter jejuni CJ677CC521	
GCF_001506845.1	Campylobacter jejuni CJ677CC523	
GCF_001506725.1	Campylobacter jejuni CJ677CC014	
GCF_001507225.1	Campylobacter jejuni CJ677CC086	
GCF_001506945.1	Campylobacter jejuni CJ677CC533	
GCF_001507245.1	Campylobacter jejuni CJ677CC095	
GCF_001507265.1	Campylobacter jejuni CJ677CC012	
GCF_001506585.1	Campylobacter jejuni CJ677CC032	
GCF_001507105.1	Campylobacter jejuni CJ677CC541	
GCF_001506265.1	Campylobacter jejuni CJ677CC073	WP_079756629.1
GCF_001506405.1	Campylobacter jejuni CJ677CC041	
GCF_001506605.1	Campylobacter jejuni CJ677CC033	WP_059119230.1
GCF_001506645.1	Campylobacter jejuni CJ677CC542	
GCF_001506765.1	Campylobacter jejuni CJ677CC085	

GCF_001506785.1	Campylobacter jejuni CJ677CC052			
GCF_001506865.1	Campylobacter jejuni CJ677CC540			
GCF_001506925.1	Campylobacter jejuni CJ677CC539			
GCF_001507205.1	Campylobacter jejuni CJ677CC034			
GCF_003368245.1	Campylobacter jejuni CJ066CC508			
GCF_000934305.1	Campylobacter jejuni subsp. Jejuni 35925			WP_044305740.1
GCF_002024325.1	Campylobacter jejuni NCTC12662		WP_079005337.1	WP_044305740.1
GCF_002028305.1	Campylobacter jejuni subsp. Jejuni ATCC 35925	WP_079399331.1	WP_079005337.1	WP_044305740.1
GCF_003060765.1	Campylobacter jejuni NCTC 12661		WP_079005337.1	WP_044305740.1
GCF_001951255.1	Campylobacter jejuni HF5-5-1	WP_038402013.1	WP_075888393.1	WP_075888364.1
GCF_001951275.1	Campylobacter jejuni HF5-7-1	WP_038402013.1	WP_075888393.1	WP_075888364.1
GCF_002234455.1	Campylobacter jejuni RM1246-ERRC	WP_093958665.1	WP_079467350.1	WP_087694997.1
GCF_000493495.1	Campylobacter jejuni 4031		WP_079279415.1	
GCF_003368065.1	Campylobacter jejuni CJ515CC45			
GCF_003368125.1	Campylobacter jejuni CJ513CC45			
GCF_003368085.1	Campylobacter jejuni CJ031CC45			
GCF_000017905.1	Campylobacter jejuni subsp. jejuni 81116	WP_012006626.1	WP_079254164.1	WP_012006635.1
GCF_002209065.1	Campylobacter jejuni subsp. Jejuni FDAARGOS_266	WP_012006626.1	WP_079254164.1	WP_088592094.1
GCF_003368045.1	Campylobacter jejuni CJ067CC45			
GCF_000737085.1	Campylobacter jejuni subsp. Jejuni MTVDSCj20	WP_038402013.1	WP_075888393.1	WP_014516918.1

GCF_000148705.1	Campylobacter jejuni subsp. jejuni M1			WP_075888393.1		WP_014516918.1
GCF_001412295.1	Campylobacter jejuni CJM1cam			WP_075888393.1		WP_014516918.1
GCF_003368105.1	Campylobacter jejuni CJ017CCUA					
GCF_001457695.1	Campylobacter jejuni NCTC11351	WP_058207847.1		WP_079754350.1		
GCF_002209005.1	Campylobacter jejuni subsp. Jejuni FDAARGOS_262	WP_058207847.1		WP_079754350.1		
GCF_001299565.1	Campylobacter jejuni subsp. Jejuni RM3197	WP_002854643.1		WP_079254326.1		WP_002854722.1
GCF_001299595.1	Campylobacter jejuni subsp. Jejuni RM3196	WP_002854643.1		WP_079254326.1		WP_002854722.1
GCF_000015525.1	Campylobacter jejuni subsp. jejuni 81-176	WP_009881803.1				WP_009881974.1
GCF_002224385.1	Campylobacter jejuni 81-176_G1_B0	WP_009881803.1				WP_009881974.1
GCF_001686905.1	Campylobacter jejuni subsp. Jejuni RM1285			WP_010891944.1		WP_065453843.1
GCF_001767215.1	Campylobacter jejuni subsp. jejuni str. RM3420	WP_070363962.1		WP_079461914.1		WP_002851876.1
GCF_002214785.1	Campylobacter jejuni FORC_046	WP_052791709.1				WP_002851876.1
GCF_001717625.1	Campylobacter jejuni subsp. Jejuni 14980A		WP_079460008.1	WP_079851618.1		
GCF_003368205.1	Campylobacter jejuni CJ090CC1332					
GCF_003368225.1	Campylobacter jejuni CJ018CCUA		WP_115766720.1			
GCF_003368185.1	Campylobacter jejuni CJ071CC464					
GCF_001865435.1	Campylobacter jejuni IF1100					
GCF_000835285.1	Campylobacter jejuni subsp. Jejuni 00-1597		WP_079394622.1	WP_079394630.1		WP_042635812.1
GCF_001865595.1	Campylobacter jejuni subsp. jejuni M129	WP_014517177.1	WP_079460163.1	WP_079865828.1		
GCF_001865395.1	Campylobacter jejuni FJ3124		WP_079460008.1			

GCF_003368145.1	Campylobacter jejuni CJ088CC52					
GCF_000171795.2	Campylobacter jejuni CG8421		WP_079343735.1			
GCF_000184205.1	Campylobacter jejuni subsp. jejuni S3	WP_014517177.1	WP_079254342.1	WP_079254358.1		WP_014517176.1
GCF_000011865.1	Campylobacter jejuni RM1221	WP_011049652.1		WP_079254358.1		WP_011049651.1
GCF_002587225.1	Campylobacter jejuni FDAARGOS_421	WP_011049652.1		WP_079254358.1		WP_011049651.1
GCF_003368165.1	Campylobacter jejuni CJ074CC443			WP_115769683.1		
GCF_001563565.1	Campylobacter jejuni RM3194	WP_052816913.1	WP_079460008.1	WP_010891944.1		WP_061099736.1
GCF_001721965.1	Campylobacter jejuni subsp. Jejuni MTVDSCj13		WP_079854565.1	WP_079854575.1		
GCF_001721985.1	Campylobacter jejuni subsp. Jejuni MTVDSCj16		WP_079854703.1	WP_079854727.1		
GCF_003030185.1	Campylobacter jejuni CFSAN054107		WP_107131175.1	WP_107131164.1		
GCF_001865415.1	Campylobacter jejuni TS1218		WP_071311218.1			
GCF_001870085.1	Campylobacter jejuni YQ2210					
GCF_001870105.1	Campylobacter jejuni ZP3204					
GCF_000430385.1	Campylobacter jejuni 32488			WP_079269902.1	WP_079269906.1	WP_009881974.1
GCF_002407125.1	Campylobacter jejuni CFSAN032806	WP_096777911.1		WP_096777910.1		WP_009881974.1
GCF_000772225.1	Campylobacter jejuni subsp. jejuni F38011	WP_009881803.1		WP_010891944.1		WP_041160003.1
GCF_002209045.1	Campylobacter jejuni subsp. Jejuni FDAARGOS_265		WP_079460163.1	WP_088585164.1		
GCF_001587015.1	Campylobacter jejuni OD267			WP_061577918.1		
GCF_001587035.1	Campylobacter jejuni WP2202					WP_061563569.1
GCF_000302555.5	Campylobacter jejuni subsp. jejuni PT14		WP_079768733.1	WP_079768734.1		WP_015016423.1

GCF_001721945.1	Campylobacter jejuni subsp. Jejuni MTVDSCj07	WP_069358644.1		WP_010891944.1		WP_015016423.1
GCF_001314285.1	Campylobacter jejuni RM1285			WP_010891944.1		
GCF_003060785.1	Campylobacter jejuni NCTC 12664			WP_108614575.1		WP_108614536.1
GCF_001951235.1	Campylobacter jejuni HF5-4A-4		WP_075912927.1	WP_075912974.1		WP_075912928.1
GCF_002587105.1	Campylobacter jejuni FDAARGOS_422	WP_002851724.1		WP_010891944.1		WP_002851876.1
**GCF_000009085.1**	**Campylobacter jejuni subsp. jejuni NCTC 11168 = ATCC 700819**	**YP_002343604.1**		**YP_002344933.1**		**YP_002343704.1**
GCF_000304375.1	Campylobacter jejuni subsp. jejuni NCTC 11168-BN148	WP_009881803.1		WP_010891944.1		WP_009881974.1
GCF_000830775.1	Campylobacter jejuni subsp. jejuni NCTC 11168-K12E5	WP_041337137.1		WP_079394646.1		WP_041337140.1
GCF_000830805.1	Campylobacter jejuni subsp. jejuni NCTC 11168-Kf1	WP_041337137.1		WP_079394646.1		WP_041337140.1
GCF_000830825.1	Campylobacter jejuni subsp. jejuni NCTC 11168-mcK12E5	WP_009881803.1		WP_010891944.1		WP_009881974.1
GCF_000830845.1	Campylobacter jejuni subsp. jejuni NCTC 11168-mfK12E5	WP_009881803.1		WP_010891944.1		WP_009881974.1
GCF_000830865.1	Campylobacter jejuni subsp. jejuni NCTC 11168-GSv	WP_009881803.1		WP_010891944.1		WP_009881974.1
GCF_002209025.1	Campylobacter jejuni subsp. Jejuni FDAARGOS_263	WP_009881803.1				WP_009881974.1
GCF_002224325.1	Campylobacter jejuni 11168H/lacY	WP_009881803.1		WP_010891944.1		WP_009881974.1
GCF_002238375.1	Campylobacter jejuni 11168H/araE	WP_009881803.1		WP_010891944.1		WP_009881974.1
GCF_900475265.1	Campylobacter jejuni subsp. jejuni	WP_009881803.1		WP_010891944.1		WP_009881974.1
GCF_003060745.1	Campylobacter jejuni NCTC 12660			WP_108592625.1	WP_108592594.1	
GCF_001951295.1	Campylobacter jejuni NS4-1-1			WP_072223305.1	WP_075867322.1	
GCF_001951315.1	Campylobacter jejuni NS4-5-1			WP_072223305.1	WP_075867322.1	
GCF_001951335.1	Campylobacter jejuni NS4-9-1			WP_072223305.1	WP_075867322.1	

	GCF_000025425.1	Campylobacter jejuni subsp. jejuni IA3902			WP_014516860.1	WP_079254169.1	
	GCF_000835345.1	Campylobacter jejuni subsp. Jejuni 01-1512		WP_079394712.1	WP_079394745.1		WP_009881974.1
	GCF_000835365.1	Campylobacter jejuni subsp. Jejuni 00-0949		WP_079394712.1	WP_079394721.1		
	GCF_003060725.1	Campylobacter jejuni 12567					
	GCF_002101355.1	Campylobacter jejuni YH002			WP_014516860.1	WP_079254169.1	
	GCF_000835305.1	Campylobacter jejuni subsp. Jejuni 00-6200			WP_014516860.1	WP_079394740.1	
	GCF_000466075.2	Campylobacter jejuni subsp. jejuni 00-2544			WP_079376548.1	WP_079376546.1	
	GCF_000466065.2	Campylobacter jejuni subsp. jejuni 00-2538			WP_079376548.1	WP_079376546.1	
	GCF_000466105.2	Campylobacter jejuni subsp. jejuni 00-2426			WP_079376548.1	WP_079376546.1	
	GCF_000468915.2	Campylobacter jejuni subsp. jejuni 00-2425			WP_079376548.1	WP_079376546.1	
Campylobacter_D cuniculorum	GCF_002104335.1	Campylobacter cuniculorum DSM 23162 = LMG 24588					
	GCF_000621005.1	Campylobacter cuniculorum DSM 23162 = LMG 24588					
Campylobacter_D helveticus	GCF_002080395.1	Campylobacter helveticus	WP_082200713.1				WP_082200691.1, WP_082200520.1
	GCF_900176295.1	Campylobacter helveticus					
Campylobacter_D upsaliensis	GCF_000620965.1	Campylobacter upsaliensis DSM 5365					
	GCF_000167395.1	Campylobacter upsaliensis RM3195	WP_004275314.1				
	GCF_000185345.1	Campylobacter upsaliensis JV21					
Campylobacter_D sp002738235	GCF_002738235.1	Campylobacter sp. 73/13					

	GCF_002738305.1	Campylobacter sp. 251/13			
Campylobacter_D hepaticus	GCF_001687475.1	Campylobacter hepaticus			
Campylobacter_D coli	GCF_000254135.1	Campylobacter coli LMG 9860			
	GCF_002024185.1	Campylobacter coli aerotolerant OR12		WP_099046908.1	
	GCF_000465235.1	Campylobacter coli CVM N29710		WP_021033641.1	
	GCF_000494775.1	Campylobacter coli 15-537360	WP_023362248.1	WP_023362235.1	
	GCF_000583755.1	Campylobacter coli RM1875			
	GCF_000583795.1	Campylobacter coli RM5611	WP_025431530.1		WP_025431485.1
	GCF_000954195.1	Campylobacter coli FB1	WP_045243712.1		WP_045243711.1
	GCF_001417635.1	Campylobacter coli HC2-48			
	GCF_001483845.1	Campylobacter coli OR12		WP_058914555.1	
	GCF_001639125.1	Campylobacter coli YH501		WP_063651861.1	
	GCF_001717605.1	Campylobacter coli 14983A		WP_057992610.1	
	GCF_001865495.1	Campylobacter coli BP3183		WP_071304885.1	
	GCF_001865515.1	Campylobacter coli WA333			
	GCF_001865555.1	Campylobacter coli BG2108			
	GCF_001936355.1	Campylobacter coli YH502		WP_075395263.1	
	GCF_002407145.1	Campylobacter coli CFSAN032805		WP_096788361.1	
	GCF_002843985.1	Campylobacter coli YH503		WP_101348801.1	

	GCF_003030205.1	Campylobacter coli CFSAN054106	WP_107128010.1	WP_107128017.1			
	GCF_001417615.1	Campylobacter coli CO2-160	WP_060473514.1				
	GCF_001417655.1	Campylobacter coli CF2-75					
Campylobacter_D coli_A	GCF_000470055.1	Campylobacter coli 76339	WP_020973958.1				
	GCF_000470055.1	Campylobacter coli	WP_020973958.1				
	GCF_001488335.1	Campylobacter coli					
	GCF_002019885.1	Campylobacter coli VA7		WP_078397504.1			
	GCF_001495535.1	Campylobacter coli					
	GCF_001498475.1	Campylobacter coli		WP_075504589.1			
	GCF_001494895.1	Campylobacter coli		WP_075482578.1			
Campylobacter_D coli_B	GCF_001492295.1	Campylobacter coli		WP_058968202.1			
	GCF_002019985.1	Campylobacter coli VA46		WP_075447212.1			
	GCF_002020085.1	Campylobacter coli VA37					
	GCF_002019865.1	Campylobacter coli VA6		WP_078416234.1			
	GCF_002020015.1	Campylobacter coli VA8		WP_075447212.1			WP_052774443.1
	GCF_001491515.1	Campylobacter coli		WP_075438466.1			
	GCF_001490995.1	Campylobacter coli					
	GCF_001497615.1	Campylobacter coli		WP_075457517.1			

Table S3: Glycan structures recognized by (A) $Tlp2^{LBD}$, (B) $Tlp3^{LBD}$ and (C) $Tlp4^{LBD}$. Blood group A antigens -A (typeX), Blood group B antigens -B (typeX), Blood group H antigens -H (typeX), $Lewis^{a}$ (Le^{a}), $Lewis^{b}$ (Le^{b}), $Lewis^{c}$ (Le^{c}), $Lewis^{x}$ (Le^{x}), $Lewis^{y}$ (Le^{y}), Ganglioside asialo GM1(Asialo-GM1), Thomsen-Friedenreich (TF) antigens, and Thomsen nouvelle (Tn) antigens. GM1(Asialo-GM1), Thomsen-Friedenreich (TF) antigens, P antigens P_1, P_2, P^k, and P and Thomsen nouvelle (Tn) antigens.

(A) **Tlp2**

Glycan No	Structure	Common name
240	(Glcα1-4)3β-sp4	TRISACCHARIDES
390	(Glcα1-4)4β-sp4	TRISACCHARIDES
241	(Glcα1-6)3β-sp4	TRISACCHARIDES
492	(Glcα1-6)5β-sp4	PENTA-NONA SACCHARIDES
502	(Glcα1-6)6β-sp4	PENTA-NONA SACCHARIDES
165	GlcAβ1-3Galβ-sp3	DISACCHARIDES
164	GlcAβ1-3GlcNAcβ-sp3	DISACCHARIDES
166	GlcAβ1-6Galβ-sp3	DISACCHARIDES
392	Fucα1-2(GalNAcα1-3)Galβ1-3GalNAcα-sp3	A (type 3)
366	Fucα1-2(GalNAcα1-3)Galβ1-3GlcNAcβ-sp3	A (type 1)
368	Fucα1-2(GalNAcα1-3)Galβ1-4GlcNAcβ-sp3	A (type 2)
363	Fucα1-2(Galα1-3)Galβ1-3GalNAcβ-sp3	B (type 4)
359	Fucα1-2(Galα1-3)Galβ1-3GlcNAcβ-sp3	B (type 1)
360	Fucα1-2(Galα1-3)Galβ1-4GlcNAcβ-sp3	B (type 2)
371	Fucα1-2Galβ1-3(Fucα1-4)GlcNAcβ-sp3	Le^{b}
480	Fucα1-2Galβ1-3GlcNAcβ1-3Galβ1-4GlcNAcβ-sp2	H (type1) penta
19L	Fucα1-2Galβ1-4(Fucα1-3)GlcNAcβ1-3Gal	Le^{y} pentaose
216	Fucα1-2Galβ1-4GlcNAcβ-sp3	H (type 2)
219	Fucα1-2Galβ1-4Glcβ-sp4	H (type 6)
71	Fucα1-2Galβ-sp3	H_{di}
483	Fucα1-3(Fucα1-2 (Galα1-3)Galβ1-4)GlcNAcβ-sp3	Ble^{y}
497	Fucα1-3(Fucα1-2Galβ1-4)GlcNAcβ1-3Galβ1-4Glcβ-sp4	Le^{y}-Lac
372	Fucα1-3(Fucα1-2Galβ1-4)GlcNAcβ-sp3	Le^{y}
364	Fucα1-3(Galα1-3Galβ1-4)GlcNAcβ-sp3	αGal-Le^{x}
234	Fucα1-3(Galβ1-4)GlcNAcβ-sp3	Le^{x}
429	Fucα1-3(Neu5Acα2-3(6-O-Su)Galβ1-4)GlcNAcβ-sp3	TETRASACCHARIDES
428	Fucα1-3(Neu5Acα2-3Galβ1-4)6-O-Su-GlcNAcβ-sp3	TETRASACCHARIDES
423	Fucα1-3(Neu5Acα2-3Galβ1-4)GlcNAcβ-sp3	Sia-Le^{x}
18E	GalNAcα1-3(Fucα1-2)Galβ1-4(Fucα1-3)Glc	Blood group A pentasaccharide
9A	Galα1-3(Fucα1-2)Galβ1-4(Fucα1-3)Glc	Blood Group B pentasaccharide
233	Galβ1-3(Fucα1-4)GlcNAcβ-sp3	Le^{a}

8C	Galβ1-3GlcNAcβ1-3Galβ1-4(Fucα1-3)GlcNAcβ1-3Galβ1-4Glc	Fucosylated glycans
19J	Galβ1-4(Fucα1-3)GlcNAcβ1-3Gal	Le^x tetraose
8E	Galβ1-4(Fucα1-3)GlcNAcβ1-6(Fucα1-2Galβ1-3GlcNAcβ1-3)Galβ1-4Glc	Fucosylated glycans
289	Galα1-3(Neu5Acα2-6)GalNAcα-sp3	6-Sia-TF
2A	Galα1-3Galβ1-4Galα1-3Gal	Terminal galactose
373	Galα1-3Galβ1-4GlcNAcβ1-3Galβ-sp3	Galili 4
220	Galα1-3Galβ1-4Glcβ-sp2	TRISACCHARIDES
375	Galα1-4GlcNAcβ1-3Galβ1-4GlcNAcβ-sp3	TETRASACCHARIDES
228	Galβ1-2Galα1-4GlcNAcβ-sp4	TRISACCHARIDES
145	Galβ1-3(6-O-Su)GlcNAcβ-sp3	6-O-Su-Le^c
388	Galβ1-3(Galβ1-4GlcNAcβ1-6)GalNAcα-sp3	TETRASACCHARIDES
382	Galβ1-3GalNAcβ1-4Galβ1-4Glcβ-sp3	Asialo-GM1
229	Galβ1-3Galβ1-4GlcNAcβ-sp4	TRISACCHARIDES
377	Galβ1-3GlcNAcβ1-3Galβ1-3GlcNAcβ-sp2	TETRASACCHARIDES
379	Galβ1-3GlcNAcβ1-3Galβ1-4GlcNAcβ-sp3	TETRASACCHARIDES
376	Galβ1-3GlcNAcβ1-3Galβ1-4Glcβ-sp4	TETRASACCHARIDES
381	Galβ1-3GlcNAcβ1-6Galβ1-4GlcNAcβ-sp2	TETRASACCHARIDES
18M	Galβ1-4Gal	Terminal galactose
264	Galβ1-4Galβ1-4GlcNAc-sp3	TRISACCHARIDES
94	Galβ1-4Galβ-sp4	TRISACCHARIDES
488	Galβ1-4GlcNAcβ1-3(Galβ1-4GlcNAcβ1-6)GalNAcα-sp3	LN2-3,6-Tn
499	Galβ1-4GlcNAcβ1-3(Galβ1-4GlcNAcβ1-6)Galβ1-4GlcNAc-sp2	PENTA-NONA SACCHARIDES
489	Galβ1-4GlcNAcβ1-3(GlcNAcβ1-6)Galβ1-4GlcNAc-sp2	PENTA-NONA SACCHARIDES
383	Galβ1-4GlcNAcβ1-3Galβ1-4Glcβ-sp2	TETRASACCHARIDES
97	Galβ1-4GlcNAcβ-sp3	DISACCHARIDES
119	Manα1-2Manβ-sp4	DISACCHARIDES
495	Manα1-3(Manα1-3(Manα1-6)Manα1-6)Manβ-sp4	PENTA-NONA SACCHARIDES
258	Manα1-3(Manα1-6)Manβ-sp4	TRISACCHARIDES
120	Manα1-3Manβ-sp4	DISACCHARIDES
121	Manα1-4Manβ-sp4	DISACCHARIDES
5F	Manα1-6Man	Mannosyl containing glycans
122	Manα1-6Manβ-sp4	DISACCHARIDES
123	Manβ1-4GlcNAcβ-sp4	DISACCHARIDES
19F	Neu5Acα2-6Galβ1-4GlcNAcβ1-2Manα1-3(Neu5Acα2-6Galβ1-4GlcNAcβ1-2Manα1-6)Manβ1-4GlcNAcβ1-4(Fucα1-6)GlcNAc	Complex type X-glycan

(B) **Tlp3**

Glycan	Structure	Common name
12P	ΔUA-GalNAc-4S,6S	Glycosaminoglycans - high and low molecular weight
18I	GlcA	D-Glucuronic acid
12F	Neocarradecaos-41, 3, 5, 7, 9-penta-O-sulphate (Na+)	Glycosaminoglycans - high and low molecular weight

12J	ΔUA-2S-GlcNAc	Glycosaminoglycans - high and low molecular weight
13A	ΔUA-2S-GalNAc-4S	Glycosaminoglycans - high and low molecular weight
13M	(GlcA/IdoAβ1-3(±6S)Gal*N*Acβ1-4)n (n<250)	Glycosaminoglycans - high and low molecular weight
14E	HA 30000	Glycosaminoglycans - high and low molecular weight
13M	HA 107000	Glycosaminoglycans - high and low molecular weight
1N	Galα1-3Gal (α1-3 Galactobiose)	Terminal galactose
2B	Galβ1-6Gal (beta1-6galactobiose)	Terminal galactose
5G	Manα1-6(Manα1-3)Man(α1-3,α1-6-Mannobiose)	Mannosyl containing glycans
10E	Neu5Acα2-3Galβ1-3(Neu5Acα2-6)GalNAc	Sialylated glycans
8B	SO_3-3Galβ1-4(Fucα1-3)Glc*N*Ac	Fucosylated glycans
19M	Galβ1-3(Fucα1-4)GlcNAcβ1-3Gal	Le^a tetraose
19N	Fucα1-2Galβ1-3(Fucα1-4)Glc*N*Ac	Le^b tetraose
2C	GalNAcβ	Terminal galactose
20	Rhaα	MONOSACCHARIDES
19	ManNAcβ	MONOSACCHARIDES
78	Galα1-3GalNAcα-sp3	DISACCHARIDES
168	GlcNAcβ1--[HOOC(CH_3)CH]-3-O-GlcNAcβ-L-alanyl-D-i-glutaminyl-L-lysine	DISACCHARIDES
177	Galβ1-4(6-O-Su)GlcNAcβ	DISACCHARIDES
123	Manβ1-4GlcNAcβ	DISACCHARIDES
206	Neu5Gcα2-3Gal	DISACCHARIDES
232	Galβ1-4GlcNAcβ1-6GalNAcα	TRISACCHARIDES
240	$(Glcα1\text{-}4)_3β$	TRISACCHARIDES
293	Neu5Acα2-3Galβ1-4Glcβ	TRISACCHARIDES
331	Neu5Gcα2-3Galβ1-3GlcNAcβ	3`Sia-Le^c (Gc)
359	Fucα1-2(Galα1-3)Galβ1-3GlcNAcβ	B (type 1)
389	GalNAcβ1-3Galα1-4Galβ1-4Glcβ	P antigen
495	Manα1-3(Manα1-3(Manα1-6)Manα1-6)Manβ	PENTA-NONA SACCHARIDES
534	Neu5Acα2-3Galβ1-4GlcNAcβ1-3Galβ1-4GlcNAcβ	PENTA-NONA SACCHARIDES

(C) **Tlp4**

Glycan	Structure	Common name
240	(Glcα1-4)3β-sp4	TRISACCHARIDES
241	(Glcα1-6)3β-sp4	TRISACCHARIDES
45	GlcAβ-sp3	MONOSACCHARIDES
19P	Glcα1-4Glcα1-4Glcα1-4Glc	Terminal Glucose structures
110	Glcα1-4Glcβ-sp3	DISACCHARIDES
7	Glcα-sp3	MONOSACCHARIDES
112	Glcβ1-6Glcβ-sp4	DISACCHARIDES
9	Glcβ-sp3	MONOSACCHARIDES
287	3-O-Su-Galβ1-3(Fucα1-4)GlcNAcβ-sp3	Su-Le^a
8J	Fuca1-2Galb1-4(Fuca1-3)GlcNAcb1-3(Fuca1-2)Galb1-4Glc	Fucosylated glycans

366	Fucα1-2(GalNAcα1-3)Galβ1-3GlcNAcβ-sp3	A (type 1)
368	Fucα1-2(GalNAcα1-3)Galβ1-4GlcNAcβ-sp3	A (type 2)
363	Fucα1-2(Galα1-3)Galβ1-3GalNAcβ-sp3	B (type 4)
359	Fucα1-2(Galα1-3)Galβ1-3GlcNAcβ-sp3	B (type 1)
226	Fucα1-2(Galα1-3)Galβ-sp3	B_{tri}
371	Fucα1-2Galβ1-3(Fucα1-4)GlcNAcβ-sp3	Le^b
217	Fucα1-2Galβ1-3GalNAcα-sp3	H (type 3)
219	Fucα1-2Galβ1-4Glcβ-sp4	H (type 6)
288	Fucα1-3(3-O-Su-Galβ1-4)GlcNAcβ-sp3	Su-Le^x
372	Fucα1-3(Fucα1-2Galβ1-4)GlcNAcβ-sp3	Le^y
364	Fucα1-3(Galα1-3Galβ1-4)GlcNAcβ-sp3	αGal-Le^x
234	Fucα1-3(Galβ1-4)GlcNAcβ-sp3	Le^x
72	Fucα1-3GlcNAcβ-sp3	DISACCHARIDES
1	Fucα-sp3	MONOSACCHARIDES
7M	Galβ1-3(Fucα1-2)Gal	Blood Group B Trisaccharide
7J	Galβ1-3(Fucα1-4)GlcNAc	Le^a
7E	Galβ1-3(Fucα1-4)GlcNAcβ1-3Galβ1-4(Fucα1-3)Glc	Fucosylated glycans
233	Galβ1-3(Fucα1-4)GlcNAcβ-sp3	Le^a
8C	Galβ1-3GlcNAcβ1-3Galβ1-4(Fucα1-3)GlcNAcβ1-3Galβ1-4Glc	Fucosylated glycans
7H	Galβ1-4(Fucα1-3)Glc	Fucosylated glycans
7I	Galβ1-4(Fucα1-3)GlcNAc	Le^x
8F	Galβ1-4(Fucα1-3)GlcNAcβ1-6(Fucα1-2Galβ1-3(Fucα1-4)GlcNAcβ1-3)Galβ1-4Glc	Fucosylated glycans
8D	Galβ1-4(Fucα1-3)GlcNAcβ1-6(Galβ1-3GlcNAcβ1-3)Galβ1-4Glc	Fucosylated glycans
19A	Galβ1-4GlcNAcβ1-2Manα1-3(Galβ1-4GlcNAcβ1-2Manα1-6Man)β1-4GlcNAcβ1-4(Fucα1-6)GlcNAc	Complex type N-glycans
8G	Galβ1-4GlcNAcβ1-3Galβ1-4(Fucα1-3)Glc	Fucosylated glycans
8E	Galβ1-4(Fucα1-3)GlcNAcβ1-6(Fucα1-2Galβ1-3GlcNAcβ1-3)Galβ1-4Glc	Fucosylated glycans
75	Galα1-2Galβ-sp3	DISACCHARIDES
289	Galα1-3(Neu5Acα2-6)GalNAcα-sp3	TRISACCHARIDES
78	Galα1-3GalNAcα-sp3	DISACCHARIDES
77	Galα1-3GalNAcβ-sp3	DISACCHARIDES
2E	Galα1-4Galβ1-4GlcNAc	P1 antigen
224	Galα1-4Galβ1-4Glcβ-sp3	P^k antigen
375	Galα1-4GlcNAcβ1-3Galβ1-4GlcNAcβ-sp3	TETRASACCHARIDES
81	Galα1-4GlcNAcβ-sp3	DISACCHARIDES
83	Galα1-6Glcβ-sp4	DISACCHARIDES
2	Galα-sp3	MONOSACCHARIDES
84	Galβ1-2Galβ-sp3	DISACCHARIDES
254	Galβ1-3(GlcNAcβ1-6)GalNAcα-sp3	TRISACCHARIDES
1E	Galβ1-3GalNAc	Terminal galactose
89	Galβ1-3GalNAcα-sp3	DISACCHARIDES
88	Galβ1-3GalNAcβ-sp3	DISACCHARIDES
229	Galβ1-3Galβ1-4GlcNAcβ-sp4	TRISACCHARIDES
87	Galβ1-3Galβ-sp3	DISACCHARIDES
85	Galβ1-3GlcNaAcβ-sp3	DISACCHARIDES

1A	Galβ1-3GlcNAc	Terminal galactose
378	Galβ1-3GlcNAcα1-3Galβ1-4GlcNAcβ-sp3	TETRASACCHARIDES
377	Galβ1-3GlcNAcβ1-3Galβ1-3GlcNAcβ-sp2	TETRASACCHARIDES
2G	Galβ1-3GlcNAcβ1-3Galβ1-4GlcNAcβ1-6(Galβ1-3GlcNAcβ1-3)Galβ1-4Glc	Terminal galactose
1C	Galβ1-4Gal	Terminal galactose
264	Galβ1-4Galβ1-4GlcNAc-sp3	TRISACCHARIDES
19B	Galβ1-4GlcNAcβ1-2(Galβ1-4GlcNAcβ1-4)Manα1-3(Galβ1-4GlcNAcβ1-2(Galβ1-4GlcNAcβ1-6)Manα1-6Man)β1-4GlcNAcβ1-4GlcNAc	Complex type N-glycans
231	Galβ1-4GlcNAcβ1-3GalNAcα-sp3	LN3-Tn
232	Galβ1-4GlcNAcβ1-6GalNAcα-sp3	LN6-Tn
93	Galβ1-4Glcβ-sp4	Terminal galactose
2B	Galβ1-6Gal	Terminal galactose
1D	Galβ1-6GlcNAc	Terminal galactose
3	Galβ-sp3	MONOSACCHARIDES
119	Manα1-2Manβ-sp4	DISACCHARIDES
120	Manα1-3Manβ-sp4	DISACCHARIDES
20	Rhaα-sp3	MONOSACCHARIDES

Table S4. Tlp3 docking analysis.

Kd [uM]	Arg	Asp	Fu	Gal	GlcN	Glu	Iso	Lys	Mal	Man	Pur	Sia	Thia
Cluster A **(ASN 116)**	143	469	109	110	98	93	145	248	376	69	399	19	11
Cluster B **(ARG 69)**	-	851	-	-	-	-	548	574	472	-	515	-	-
Cluster C **(ASN 212)**	-	-	-	-	-	-	-		663	-	552	-	76
Cluster D **(PHE 216)**	-	731	326	196	161	143	-		468	152	567	27	-
Cluster E **(GLN 174)**	-	-	-	-	250	232	-		-	146	608	72	34
Binding energy [kcal/mol]	**Arg**	**Asp**	**Fu**	**Gal**	**GlcN**	**Glu**	**Iso**	**Lys**	**Mal**	**Man**	**Pur**	**Sia**	**Thia**
Cluster A **(ASN 116)**	5.25	4.54	5.41	5.40	5.47	5.49	5.24	4.92	4.67	5.67	4.64	6.47	6.75
Cluster B **(ARG 69)**	-	4.19	-	-	-	-	4.45	4.42	4.54	-	4.49	-	-
Cluster C **(ASN 212)**	-	4.12	-	-	-	-	-		4.34	-	4.44	-	5.62
Cluster D **(PHE 216)**	-	4.28	4.76	5.06	5.17	5.24	-		4.54	5.21	4.43	6.23	-
Cluster E **(GLN 174)**	-	-	-	-	4.91	4.96	-		-	5.23	4.39	5.65	6.10
#conformers	**Arg**	**Asp**	**Fu**	**Gal**	**GlcN**	**Glu**	**Iso**	**Lys**	**Mal**	**Man**	**Pur**	**Sia**	**Thia**
Cluster A **(ASN 116)**	1000	530	780	685	550	650	950	950	350	800	400	750	650
Cluster B **(ARG 69)**	-	55	-	-	-		50	50	250	-	200	-	-
Cluster C **(ASN 212)**	-	-	-	-	-		-	-	50	-	150	-	50
Cluster D **(PHE 216)**	-	315	120	315	400	300	-	-	250	150	150	200	-
Cluster E **(GLN 174)**	-	-	-	-	50	50	-	-	-	50	50	50	300

Table S5: Summary of chemotactic responses of Δ*tlp3*, Δ*tlp2*, Δ*tlp4*, Δ*tlp2,4*, Δ*tlp2,3*, Δ*tlp3,4* and Δ*tlp2,3,4* strains illustrated as log-changes compared to the wild type. Green indicates ligands identified as chemoattractants (down), red indicates ligands identified as chemorepellents (UP) and yellow indicates ligands that showed no significant difference compared with wild-type strain (*P*<0.05).

Ligands	***Δtlp3 ****	***Δtlp2***	***Δtlp4***	***Δtlp2,4***	***Δtlp2,3***	***Δtlp3,4***	***Δtlp2,3,4***
	Depletion assay*	Depletion assay	Depletion assay	Depletion assay	Depletion assay	Depletion assay	Depletion assay
Serine		2.0 down	1.5 down	2.7 down	2.2 down	1.5 down	3.1 down
Methionine		1.8 down	1.4 down	1.9 down	1.9 down	2.2 down	2.0 down
Asparagine		1.7 down	2.4 down	2.7 down	1.7 down	2.3 down	3.5 down
Cysteine		1	1.4 down	1.4 down	1	1.3 down	1.9 down
Lysine	1.6 UP	1.4 UP	1.8 down	1.1 down	1.8 UP	1	1.5 UP
Purine	1.2 down	1.2 down	1.1 down	1.4 down	1.4 down	1.3 down	2.6 down
Aspartate	1.9 down	1.7 down	1.1 down	1.7 down	2.3 down	1.8 down	2.9 down
Isoleucine	2.7 down						
Malic acid	2.0 down						
Fumaric acid	2.2 down						
Arginine	1.3 UP						
Glucosamine	2.1 UP						
Thiamine	1.2 UP						
Succinic acid	1.2 UP						
**Glucose**	1.2 down	1.6 down	1.4 down	1.7 down	1.4 down	1.2 down	2.4 down
**Fucose**	1.4 down	1.7 down	1.4 down	2.4 down	2.1 down	2.0 down	3.4 down
**Galactose**	1	1	1	1.1 down	1.1 down	1.1 down	1.4 down
**Mannose**	1.4 UP	1.2 down	1.0 down	1.4 down	1.2 UP	1.3 UP	1.2 UP
**Sialic acid**	1.2 UP	1.4 down	1.2 down	1.3 down	1.1 UP	1	1.1 down

Table S6. Microbial strains and plasmids

Strain/plasmid	Description	Reference/source
Bacterial strains/ plasmids		
C. jejuni 11168-O	Wild type (human isolated strain) originally strain 5636/77	[1])
C. jejuni 81116 Δ*flaA⁻/flab⁻*	Isogenic mutant of *flaA/flaB*	J.M. Ketley
C. jejuni 11168-OΔ*tlp3::aphA3*	Isogenic mutant of *tlp3*	[2]
C. jejuni 11168-OΔ*tlp2*::*Er^R*	Isogenic mutant of *tlp2*	This study
C. jejuni 11168-OΔ*tlp4::Cm^R*	Isogenic mutant of *tlp4*	This study
C. jejuni 11168-OΔ*tlp2::Er^R*Δ*tlp4::Cm^R*	Isogenic mutant of *tlp2* and *tlp4*	This study
C. jejuni 11168-OΔ*tlp2::Er^R*Δ*tlp3::Km^R*	Isogenic mutant of *tlp2* and *tlp3*	This study
C. jejuni 11168-OΔ*tlp3::Km^R*Δ*tlp4::Cm^R*	Isogenic mutant of *tlp3* and *tlp4*	This study
C. jejuni 11168-OΔ*tlp2::Er^R*Δ*tlp3::Km^R*Δ*tlp4::Cm^R*	Isogenic mutant of *tlp2, tlp3* and *tlp4*	This study
C. jejuni 11168-OΔ*tlp2*::Er^RΩ*tlp2*::Km	Complemented *tlp2*$^{-/+}$ mutant in *C. jejuni 11168-OΔtlp2::Er^R*	This study
C. jejuni 11168-OΔ*tlp4*::Cm^RΩ*tlp4*::Km	Complemented *tlp4*$^{-/+}$ mutant in *C. jejuni 11168-OΔtlp4::Cm^R*	This study
*C. jejuni*11168-OΔ*tlp3 ::aphA3 Ωtlp3::cat*	Complemented *tlp3*$^{-/+}$ mutant in *C. jejuni* 11168-OΔ*tlp3::Km*	[2,3]
E. coli BL21(DE3)	F$^-$ *ompT hsdS$_B$* (r_B^- m_B^-) *gal dcm* (DE3)	Novagen
E. coli DH5α	F$^-$, ø80d*lacZ*ΔM15, Δ(*lacZYA-argF*)U169, *deo*R, *recA*1, *endA*1, *hsdR*17(rK$^-$, mK$^+$), *phoA*, *supE*44, λ–, *thi*-1, *gyrA*96, *relA*1	[3]
pGEM-T Easy	Cloning vector	Promega
pET-19b	His-tag expression vector	Novagen
pGU0504	*tlp2^peri* cloned into pGEM-T Easy	This study
pGU0706	*tlp4^peri* cloned into pGEM-T Easy	This study
pGU0505	*tlp2^peri* cloned into pET-19b	This study
pGU0707	*tlp4^peri* cloned into pET-19b	This study
pGU0816	*tlp3^peri* cloned into pET-19b	[2]
pGU0817	Tlp3^{LBD}K149A	This study
pGU0818	Tlp3^{LBD}W151A	This study
pGU0819	Tlp3^{LBD}D169A	This study
pGU0820	Tlp3^{LBD}T170A	This study
pGU0821	Tlp3^{LBD}D196A	This study
pGU0510	Δ*tlp2*::Er^R cloned into pGEM-T Easy	This study

pGU0720	Δ*tlp4*::Cm^R cloned into pGEM-T Easy	This study
pGU0817	Δ*tlp3*::Km^R cloned into pGEM-T Easy	[2]
pK46*tlp2*	pK46:*tlp2*^{peri}	This study
pK46*tlp4*	pK46:*tlp4*^{peri}	This study
pK46	*C. jejuni cj0046* pseudogene integration vector; Km^R	[2,4]

Table S7. Primers used in this study.

Primer name	Sequence 5'-3'	Restriction Site	Reference
Tlp2^{LBD} Forward	CATATGCAAGCAATTTTTCATGAAGTTGTGA	NdeI	This study
Tlp2^{LBD} Reverse	CTCGAGTTATAAACTGGAGCTTCTATTTGTT	XhoI	This study
Tlp2 Forward	CATATGAAAAGCGTAAAATTG	NdeI	This study
Tlp2 Reverse	CTCGAGTTAAAACCTCTTCTTCTTAACATC	XhoI	This study
Tlp2_invMUT Forward	AGATCTCTGCAGCAGCTTTAGAAGAG	BglII	This study
Tlp2_invMUT Reverse	AGATCTGCCGCAAGAATATCTACAGC	BglII	This study
Tlp4^{LBD} Forward	CTCGAGGATTCGAGAAACAATACATATGAATT	XhoI	This study
Tlp4^{LBD} Reverse	CTCGAGTTGTTTCATTAAAATAGAATTAACAGC	XhoI	This study
Tlp4 Forward	GGTACCCAATCAATAAATTCAGG	KpnI	This study
Tlp4 Reverse	CTCGAGTTAAAACCTCTTCTTCTTAACATC	XhoI	This study
Tlp4_invMUT Forward	AGATCTCACCAAGGCGTAGATTATGC	BglII	This study
Tlp4_invMUT Reverse	AGATCTTTCCAACCCATAGCGTAAGC	BglII	This study
Tlp2Com Forward	ACTCGTCTCATGAAAAGCGTAAAATTG	BsmBI	This study
Tlp2Com Reverse	GTCCGTCTCTTAAAACCTCTTCTTCTTAAC	BsmBI	This study
Tlp3 Forward	ATGCTAAAAATAACAAAGATTAAAAG	-	This study
Tlp3 Reverse	AAACCTCTTCTTCTTAACATC	-	This study
Tlp4Com Forward	ACTCGTCTCATGCAATCAATAAATTCAGG	BsmBI	This study
Tlp4Com Reverse	GTCCGTCTCTTAAAACCTCTTCTTCTTAACATC	BsmBI	This study
cj0046 Forward	CTCTCTCCGCTAGAAATTAAATCC	-	This study
Ery_ Forward (ErmCBgIII-F)	AGATCTATGAACGAGAAAAATATAAAACACAG	BglII	This study
Ery Reverse (ErmCBgIII-R)	AGATCTTTACTTATTAAATAATTTATAGCTA	BglII	This study
Chlr_ Forward	AGATCTATGATGCAATTCACAAAGATT	BglII	This study
Chlr_ Reverse	AGATCTTTATTTATTCAGCAAGTCTTG	BglII	This study
Kan_ Forward	AATGGCTAAAATGAGAATATC	-	This study
Kan_ Reverse	AAACAATTCATCCAGTAAAAT	-	This study
pC46 Cat Forward	ATGATGCAATTCACAAAGATT	-	[2]
pC46 Cat Reverse	TTATTTATTCAGCAAGTCTTG	-	[2]
pK46 Kan Forward	AATGGCTAAAATGAGAATATC	-	[2]
pK46 Kan Reverse	AAACAATTCATCCAGTAAAAT	-	[2]

Table S8 - Supplementary glycan microarray document based on MIRAGE guidelines. DOI: 10.1093/glycob/cww118.

Classification	Guidelines
**1. Sample: Glycan Binding Sample**	
Description of Sample	<u>Sample names:</u><br>Tlp2,3,and 4 proteins<br><u>Origin:</u> *Helicobacter pylori* SS1*;* produced as a recombinant His -tagged protein in *E. coli*.<br><br><u>Method of preparation:</u><br>The preparation of Tlp2,3, and 4 is explained in the Materials and Methods section: Expression and purification of the periplasmic domain of Tlp2,3, and 4.
Sample modifications	Sample is a His Tagged protein containing only the periplasmic receptor domain of the full length Tlp2,3, and 4 transmembrane protein.
Assay protocol	*Please see Materials and Methods: Tlps 2/3/4 bind to a range of ligands, including glycans and amino acids*<br><br>Proteins were complexed with mouse anti-his antibody, with secondary and tertiary AlexaFluor555 antibodies (rabbit anti-mouse, goat anti-rabbit) at a molar ratio of 4:2:1. The protein/antibody complex was incubated on ice for 10 mins prior to placing on the array. Arrays were performed with 1 µg of complexed protein incubated on the array for 20 min. Arrays were then washed 3 times in Array PBS (PBS + 2mM $MgCl_2$ + 2mM $CaCl_2$) then dried by centrifugation for 3 min at 300 x *g*. Scanning was performed using an Innopsys InnoScan 1100AL and analyzed using Mapix (Innopsys). Yes/no binding was determined by six positive replicate spots in three replicate experiments. Positive binding was determined by spots being significantly greater than negative control spots by two-tailed *t* test.
**2.1 Glycan Library**	
Glycan description for defined glycans	Glycans in this study are listed in Table S2 and is a published library in doi: 10.1371/journal.pntd.0004120.
Glycan description for undefined glycans	N/A.
Glycan modifications	Glycans were prepared in one of two ways for printing:<br>1. Glycans (with IDs in number/letter format; e.g. 1A, 4C, 7K) were sourced commercially from Dextra Laboratories, Elicityl and Carbosythn and were made into glycoamines using the protocol published in Day et al 2009 (doi: 10.1371/journal.pone.0004927).

	2. Glycans (with IDs in number only format) were obtained from Prof Nicolai Bovin and were modified with spacers as per DOI: 10.1073/pnas.0407902101. The library of these glycans was first published in DOI: 10.1016/j.molimm.2009.06.010
**2.2 Small molecule Library**	
Sample description for defined compounds	Compounds printed in the amino acid/small molecule array for this study are listed in Table1 and Table S5.
**3. Printing Surface; e.g., Microarray Slide**	
Description of surface	Epoxy activated glass microarray slides.
Manufacturer	ArrayIt SuperEpoxy 3 (SME3).
Custom preparation of surface	N/A.
Non-covalent Immobilisation	N/A.
**4. Arrayer (Printer)**	
Description of Arrayer	**Glycan array:** SpotBot® Extreme Protein Microarray Spotter (ArrayIt, California, USA).<br>**Small molecule array:** ArrayJet Argus Marathon non-contact printer.
Dispensing mechanism	**Glycan array:** Contact printing using 946NS6 pins with a 6 pin in a 3 columns x 2 rows configuration.<br>**Small molecule array:** Non-contact jet printing
Glycan and small molecule deposition	**Glycan array:** Approximately 1.8 nl per spot is printed according to manufactures guidelines.<br>Glycan were at 500 µM in 50:50 DMF:DMSO.<br>**Small molecule array:** Approximately 320 nl per spot is printed according to manufactures guidelines.<br>Samples were at 1000 µM in PBS with 2% glycerol
Printing conditions	**Glycan array:** Array were printed with dehumidification at a maximum humidity of 60% relative humidity (Standard laboratory starting humidity of 75-90%) at 22ºC. Glycans were left to react with the slide for at least 8 hours after the print was completed.<br>**Small molecule array:** Array were printed with dehumidification at a maximum humidity of 60% relative humidity (Standard laboratory starting humidity of 75-90%) at 22ºC. Samples were left to react with the slide for at least 15 hours after the print was completed.
**5.1 Glycan array with “Map”**	

Array layout	The array consists of a single array of glycans split between 6 pins (3 columns x 2 rows) with 4500μm row and column spacing. Each pin printed a 20 columns x 16 rows with 200μm spot spacing (centre to centre) with a minimum spot size of 100μm. Each sample is printed in quadruplicate with each of the 6 print areas including at least three negative control samples (print solution only) and two positive control samples consisting of one sample of fluoroscienamine and one sample of a mixture of rabbit anti-mouse antibody labeled with Alexa 555 and Alexa 647. Positive controls provide proof of successful immobilization of the amine reagents and provides for orientation for analysis. The antibodies also can provide controls for secondary antibodies used in experiments (if applicable).
Glycan identification and quality control	Arrays are quality controlled by a range of measures. 1. Each printed array is post print scanned to confirm deposition of the glycans on the array surface prior to neutralization of the remaining slide surface. 2. Post neutralized slides are scanned again to monitor for remaining autofluorescence. 3. Slides are assayed with fluorescently labeled lectins: WGA-Texas Red (EY Laboratories) and ConA-FITC (EY Laboratories).
**5.2 Small molecule array with "Map"**	
Array layout	The array consists of triplicate sub-arrays of amino acids and small moleucles (24 rows x 10 columns) with 400μm spot spacing (centre to centre) with a minimum spot size of 100μm. Each sample is printed six times, with each of the 3 sub-arrays including at least six negative control samples (print solution only) and a positive control samples consisting of one a mixture of rabbit anti-mouse antibody labeled with Alexa 555 and Alexa 647. Positive controls provide proof of successful immobilization of the amine reagents and provides for orientation for analysis.
Sample identification and quality control	Arrays are quality controlled by a range of measures. 1. Each printed array is post print scanned to confirm deposition of the samples on the array surface prior to neutralization of the remaining slide surface. 2. Post neutralized slides are scanned again to monitor for remaining autofluorescence.
**6. Detector and Data Processing**	
Scanning hardware	Inopsys InnoScan 1100AL (Lasers: 488 nM, 532 nM with two filter sets for analysis at 532 and 595 nM), 635 nM) scanner.
Scanner settings	Scanning resolution: 10μM<br>Laser channel: 532nM operating 595nM excitation / 625nM emission filter set.<br>PMT: 20% gain<br>Scan powers: Low laser power.
Image analysis software	Innopsys MAPIX.
Data processing	Data was exported as a CSV file and exported to Microsoft Excel.
**7. Glycan Microarray Data Presentation**	
Data presentation	Glycan array: Data is presented as yes/no binding in Table 1. The full list of glycans is shown in Table S5.
**8. Interpretation and Conclusion from Microarray Data**	

Data interpretation	**Glycan arrays:** We only use glycan arrays as a yes/no binding tool. Due to this we look only at binding that is unambiguously above background vs lack of binding above background. Average background + 3x standard deviation of the background of 20 sets of 4 spots of DMF:DMSO only spots is applied to determine if binding observed is significantly above background. Only spots with values equal to or greater than this value were considered as binding from data of any tested slide. These values are slide dependent.<br><br>**Small molecule arrays:** We only use amino acid arrays as a yes/no binding tool. Due to this we look only at binding that is unambiguously above background vs lack of binding above background. Average background + 3x standard deviation of the background of 6 spots of PBS + 2% glycerol only spots is applied to determine if binding observed is significantly above background. Only spots with values equal to or greater than this value were considered as binding from data of any tested slide. These values are slide dependent.
Conclusions	Tlp2, Tlp3 and Tlp4 are a paralogue family of dCache_1 chemoreceptors which capable of responding to carbohydrates including complex glycans as well as to organic and amino acids

References

1. Skirrow M (1977) Campylobacter enteritis: a" new" disease. Br Med J 2: 9-11.
2. Rahman H, King RM, Shewell LK, Semchenko EA, Hartley-Tassell LE, et al. (2014) Characterisation of a multi-ligand binding chemoreceptor CcmL (Tlp3) of *Campylobacter jejuni*. PLoS Pathog 10: e1003822.
3. Hanahan D (1983) Studies on transformation of Escherichia coli with plasmids. Journal of molecular biology 166: 557-580.
4. Gaskin D, Van Vliet A, Pearson B. The *Campylobacter* genetic toolbox: development of tractable and generally applicable genetic techniques for *Campylobacter jejuni*; 2007. BLACKWELL PUBLISHING. pp. 101-101.
